# Ratiometric Fluorescent Probes Based on Isosteviol with Identification of Maleic Acid in Starchy Foods

**DOI:** 10.3390/foods14091541

**Published:** 2025-04-28

**Authors:** Xinye Qian, Chunling Zheng, Fang Zhang

**Affiliations:** College of Food Science and Light Industry, Nanjing Tech University, Nanjing 211800, China; sdkajfas@163.com

**Keywords:** fluorescence, maleic acid, fumaric acid, isosteviol

## Abstract

The rigid saddle-shaped framework of isosteviol provides a unique host–guest recognition cavity. For the first time, we have utilized isosteviol to construct fluorescent probes 4 and 5, achieving highly selective recognition of maleic acid and fumaric acid. The experimental results indicated that neither probe 4 nor probe 5 exhibited significant fluorescence changes when exposed to fumaric acid. However, both probes demonstrated distinct ratiometric fluorescence responses upon interaction with maleic acid. For maleic acid, probes 4 and 5 showed detection limits of 4.14 × 10^−6^ M and 1.88 × 10^−6^ M, respectively. Density functional theory (DFT) calculations and ^1^H NMR spectroscopy revealed that probes 4 and 5 formed stable intermolecular hydrogen bonds with maleic acid, contributing to the observed changes in fluorescence signals. Furthermore, maleic acid was successfully detected in starch-rich dietary samples, including potatoes, sweet potatoes, and corn, utilizing the sensing capabilities of probes 4 and 5. In conclusion, probes 4 and 5 hold significant potential for the development of fluorescence-based recognition systems for fumaric acid and maleic acid.

## 1. Introduction

Cis/trans isomers are a fundamental class of stereoisomers, typically formed by the spatial arrangement of substituents in rigid molecular structures. These isomers have been extensively studied and applied across various fields, including photochemistry, materials science, insecticides, pharmaceuticals, and food additives [1]. Although cis/trans isomers share the same molecular formula, they typically exhibit distinct properties due to differences in their molecular configurations. Maleic acid and fumaric acid are key cis/trans isomers that are widely utilized in the pharmaceutical, food, and polymer industries [2,3]. Fumaric acid is a commonly used food additive, and its derivatives can be used to treat multiple sclerosis, rheumatism, psoriasis, and more [4,5,6]; maleic acid is an active inhibitor of sucrose cytidine hydrogenase and Krebs cycle glutathione coenzyme and is often used as a new acidulant in food and beverages [7]. However, the excessive accumulation of maleic acid in the human body can lead to conditions such as Fanconi syndrome, neurological disorders, cardiovascular diseases, and cancer, among others [8,9]. The widespread use of these two cis/trans isomers as food and beverage ingredients has raised significant concerns about their impacts on people’s health. Current methods for detecting cis/trans isomers mainly rely on high-performance liquid chromatography (HPLC), gas chromatography–mass spectrometry (GC–MS), ion mobility spectrometry (IMS), LC-ESIMS/MS, etc. [10,11,12]. Most of these methods are too expensive and complex to be widely used. Fluorescence sensing technology has the advantages of low cost, high sensitivity, and portability [13]. Therefore, developing fluorescent chemical sensors to detect maleic acid/fumaric acid is highly desirable.

Due to their nearly identical chemical and physical properties, distinguishing between geometric isomers can be challenging. Considerable effort has been devoted to differentiating fumaric acid from maleic acid, with numerous studies focusing on the selective detection of maleic acid. Dash et al. developed an anthracene-thiazole Schiff base probe that displayed selective and transient fluorescence enhancement upon the addition of maleic acid, while remaining unresponsive to other carboxylic acids, including malic acid, citric acid, acetic acid, cinnamic acid, and tartaric acid [14]. Kim et al. synthesized three azo-based colorimetric polymer probes (denoted as P1, P2, and P3) with varying R groups (aldehyde, thiazolidine, and nitrile) on their side chains to differentiate maleic acid from its structural analog fumaric acid through selective colorimetric sensing. P1, featuring a pendant aldehyde unit, enabled the discrimination between maleic acid and fumaric acid through a distinct color change visible to the naked eye, achieved through protonation of the β-nitrogen atom in the azo chromophore of P1 by maleic acid [15]. Goswami et al. synthesized 9-anthracenemethyl-bis(6-acetamino-2-pyridine) amine as a fluorescent probe to study its interaction with different dicarboxylic acids. Maleic acid was observed to significantly enhance the fluorescence signal (58% maximum) with a redshift in emission wavelength (10 nm), while other dicarboxylic acids (e.g., fumarate, succinic acid, etc.) had a weaker effect on fluorescence [16]. Ghosh et al. successfully synthesized two novel BINOL receptor molecules, **1** and **2**, with receptor **1** performing well in fluorescently recognizing maleic acid. Theoretical calculations (DFT/B3LYP/6-31G) show that receptor **2** leads to decreased binding capacity due to additional substituents, while receptor **1** forms a tighter hydrogen bond network with maleic acid, resulting in fluorescence quenching [17]. Samanta et al. developed a colorimetric/fluorescent probe L based on the Schiff base structure that enables highly selective differentiation of maleic acid from fumaric acid. The mechanism by which probe L recognizes maleic acid through protonation and hydrogen bonding was revealed by DFT calculations, mass spectrometry, and NMR hydrogen spectroscopy. This probe can quickly detect possible maleic acid contamination in food, which is of great significance for food safety [18]. As is well known, ratiometric fluorescent probes can provide an intrinsic calibration function for environmental interferences (e.g., solvents, impurities) by measuring fluorescence intensities at two different wavelengths [19,20]. This built-in correction reduces errors caused by variations in probe concentration and light source intensity while also expanding the dynamic range of fluorescence measurements [21]. Compared to conventional single-wavelength fluorescence methods, ratiometric fluorescence significantly enhances detection accuracy and interference resistance. Furthermore, unlike chromatographic techniques such as HPLC and GC–MS, which require extensive sample preparation and sophisticated instrumentation, ratiometric fluorescence enables rapid, real-time, and cost-effective detection, making it highly suitable for on-site applications in biological research, food safety, and environmental monitoring. However, to the best of our knowledge, the use of ratiometric fluorescent probes to discriminate between maleic acid and fumaric acid remains unexplored in this field.

**Figure 1:**
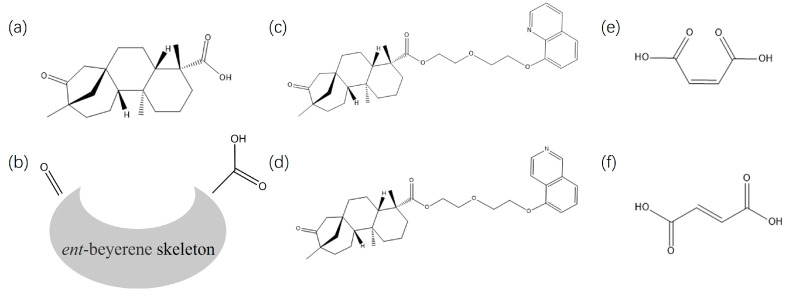
(**a**) Isosteviol, (**b**) its saddle-like structure, (**c**) probe 4 and (**d**) probe 5 based on the isosteviol, (**e**) maleic acid and (**f**) fumaric acid.

Isosteviol can be obtained through acidic hydrolysis of the natural sweetener stevia. As a natural product, isosteviol possesses multiple chiral centers and a unique rigid ent-beyerene skeleton with a saddle-like structure (Figure 1a,b) [22]. By modifying the functional groups of isosteviol, various derivatives with specific properties required for certain systems can also be synthesized [23,24]. Isosteviol has been reported to exhibit excellent applications in asymmetric catalysis, with its derivatives serving as effective catalysts for asymmetric aldol reactions [25] and Michael addition reactions [26,27]. Inspired by this, we hypothesize that this unique framework may also be applicable in molecular recognition. Compared to traditional fluorescent scaffolds, such as anthracene, rhodamine, and coumarin, isosteviol offers a unique combination of structural rigidity and biocompatibility. The rigid saddle-shaped framework of isosteviol provides a pre-organized host cavity that enhances guest selectivity and reduces non-specific binding. In addition, isosteviol is a natural derivative that can be used as a green chemical sensor material to avoid contamination by synthetic by-products when using traditional fluorescent dyes. In this study, we designed two simple and cost-effective fluorescent probes based on isosteviol (Figure 1c,d), enabling the differentiation between maleic acid and its isomer, fumaric acid (Figure 1e,f).

## 2. Materials and Methods

Stevia (A.R.), maleic acid (MA, A.R.), fumaric acid (FA, A.R.), 8-hydroxyquinoline (A.R.), and 5-hydroxyisoquinoline (A.R.) were purchased from Shanghai Macklin Biochemical Technology Co., Shanghai, China. Diethylene glycol (A.R.), p-toluensulfonyl chloride (A.R.), potassium carbonate (A.R.), and potassium hydroxide (A.R.) were purchased from Shanghai Lingfeng Chemical Reagents Co., Shanghai, China. Acetonitrile (A.R.) and dichloromethane (A.R.) were purchased from Wuxi Yasheng Chemical Co., Wuxi, China. The ^1^H NMR and ^13^C NMR spectra were obtained on a Brüker AM 400 spectrometer (Bruker Co., Karlsruhe, Germany). The HRMS spectra were obtained on a Q Exactive Benchtop Quadrupole-Orbitrap Mass spectrometer (Thermo Fisher Scientific, Waltham MA, USA). The fluorescence spectra were obtained on a Horiba FluoroMax-4 fluorescence spectrophotometer (HORIBA Ltd., Kyoto, Japan).

### 2.1. Synthesis of Probes ***4*** and ***5***

**Scheme 1:**
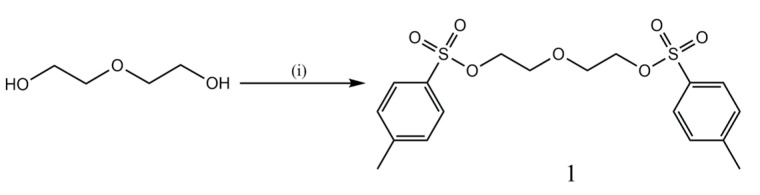
Reagent and conditions: (i) p-toluenesulfonyl chloride, CH_2_Cl_2_, ice water bath.

**Scheme 2:**
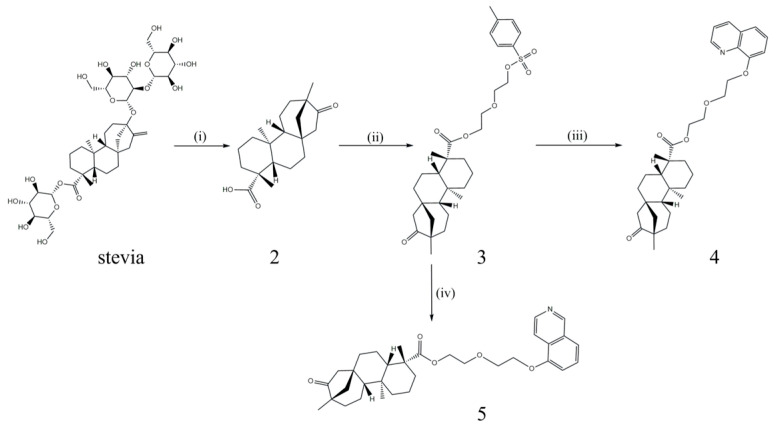
Reagent and conditions: (i) 10%H_2_SO_4_, 75 °C (ii) CH_3_CN, K_2_CO_3_, compound 1, 70 °C (iii) CH_3_CN, KOH, 80 °C (iv) CH_3_CN, K_2_CO_3_, 80 °C.

Probes **4** and **5** were synthesized according to Scheme 1 and Scheme 2. All compounds were characterized using standard methods, and data agreed with the proposed structures.

#### 2.1.1. Synthesis of Oxybis (Ethane-2,1-diyl) bis (4-Methylbenzenesulfonate)(**1**)

Compound **1** was prepared according to previous procedures outlined in the literature [28]. Diethylene glycol (2.653 g, 25 mmol) and p-toluenesulfonyl chloride (9.533 g, 50 mmol) were dissolved in methylene chloride (25 mL), and then the mixture was cooled to 0 °C using an ice water bath. Gradually, potassium hydroxide (11.22 g, 0.2 mol) was added in small increments, and subsequently, the mixture was stirred magnetically at 0 °C for 3 h. Afterward, any insoluble material was removed through suction filtration. The resulting solution was concentrated and recrystallized with methanol to yield colorless crystals of diethylene glycol bis-p-toluenesulfonate. Yield: 73%. ^1^H NMR (400 MHz, Chloroform-d) δ 7.79 (d, J = 8.4 Hz, 4H), 7.37 (d, J = 8.1 Hz, 4H), 4.23–4.02 (m, 4H), 3.73–3.58 (m, 4H), 2.46 (s, 6H).

#### 2.1.2. Synthesis of Isosteviol(**2**)

Isosteviol was prepared according to previous procedures outlined in the literature [29]. Stevia (5 g, 15.7 mmol) was dissolved in 10% sulfuric acid solution (250 mL) and reacted at 75 °C for 7 h, forming gray-white solids. The solid obtained through suction filtration was then recrystallized in low-temperature ethanol, yielding pale yellow crystals of isosteviol. Yield: 67%. ^1^H NMR (500 MHz, Chloroform-d) δ 2.64 (dd, J = 18.6, 3.8 Hz, 1H), 2.17 (dtd, J = 13.5, 3.4, 1.5 Hz, 1H), 1.93–1.33 (m, 13H), 1.25 (s, 3H), 1.23–1.13 (m, 3H), 1.03 (td, J = 13.6, 4.2 Hz, 1H), 0.98 (s, 3H), 0.92 (td, J = 13.2, 4.4 Hz, 1H), 0.79 (s, 3H).

#### 2.1.3. Synthesis of 2-(2-(Tosyloxy)ethoxy)ethyl (4R,4aS,6aR,9S,11aR,11bS)-4,9,11b-trimethyl-8-oxotetradecahydro-6a,9-methanocyclohepta[a]naphthalene-4-carboxylate(**3**)

Compound **1** (1.865 g, 4.5 mmol) and Compound **2** (0.955 g, 3 mmol) were dissolved in acetonitrile (50 mL). Potassium carbonate (0.622 g, 4.5 mmol) was then added to the mixture. The reaction was carried out at 70 °C for 12 h. After this period, the mixture was filtered to remove any solid residue. The resulting solution was concentrated to obtain a crude product, purified through column chromatography using a 3:1 mixture of petroleum ether and ethyl acetate. Yield: 89%. ^1^H NMR (400 MHz, Chloroform-d) δ 7.91–7.72 (m, 2H), 7.36 (d, J = 8.0 Hz, 2H), 4.28–4.04 (m, 4H), 3.81–3.55 (m, 4H), 2.60 (dd, J = 18.6, 3.7 Hz, 1H), 2.46 (s, 3H), 2.30–2.08 (m, 1H), 1.94–1.84 (m, 1H), 1.82 (s, 1H), 1.81–1.72 (m, 2H), 1.69 (h, J = 4.0, 3.2 Hz, 3H), 1.65 (t, J = 3.4 Hz, 1H), 1.62–1.45 (m, 3H), 1.45–1.32 (m, 3H), 1.32–1.22 (m, 2H), 1.20 (s, 3H), 1.14 (dd, J = 12.1, 2.3 Hz, 1H), 1.04 (dd, J = 13.5, 4.2 Hz, 1H), 0.99 (s, 2H), 0.91 (td, J = 13.2, 4.2 Hz, 1H), 0.69 (s, 3H).

#### 2.1.4. Synthesis of 2-(2-(Quinolin-8-yloxy)ethoxy)ethyl (4R,4aS,6aR,9S,11aR,11bS)-4,9,11b-trimethyl-8-oxotetradecahydro-6a,9-methanocyclohepta[a]naphthalene-4-carboxylate(**4**)

Compound **3** (1.4 g, 2.5 mmol) and 8-hydroxyquinoline (0.435 g, 3 mmol) were dissolved in acetonitrile (30 mL). Potassium hydroxide (0.28 g, 5 mmol) was then added to the solution, and the mixture was heated to reflux at 80 °C for 24 h. After the reaction, the precipitate was filtered out, and the resulting brown-black oily liquid was concentrated. Compound 4 was then purified using column chromatography with a solvent system of 2:1 petroleum ether and ethyl acetate. Yield: 55%. ^1^H NMR (400 MHz, Chloroform-d) δ 8.93 (dd, J = 4.2, 1.8 Hz, 1H), 8.12 (dd, J = 8.3, 1.8 Hz, 1H), 7.51–7.37 (m, 3H), 7.13 (dd, J = 7.6, 1.3 Hz, 1H), 4.42 (t, J = 5.3 Hz, 2H), 4.30–4.14 (m, 2H), 4.05 (dd, J = 5.8, 4.7 Hz, 2H), 3.83 (ddd, J = 5.7, 4.0, 1.7 Hz, 2H), 2.58 (dd, J = 18.6, 3.7 Hz, 1H), 2.23–2.10 (m, 1H), 1.92–1.30 (m, 13H), 1.26–1.05 (m, 6H), 1.04–0.92 (m, 4H), 0.85 (td, J = 13.3, 4.4 Hz, 1H), 0.67 (s, 3H). 13C NMR (101 MHz, CDCl3) δ 222.20, 177.20, 154.67, 149.29, 140.42, 135.87, 129.55, 126.68, 121.57, 120.01, 109.36, 77.34, 77.03, 76.71, 69.40, 69.29, 68.38, 63.14, 57.17, 54.73, 54.31, 48.65, 48.40, 43.83, 41.49, 39.79, 39.42, 38.00, 37.91, 37.32, 28.89, 21.61, 20.29, 19.85, 18.91, 13.25. HRMS: Calculated *m*/*z* for C_33_H_43_NO_5_ (M + H): 534.32065, found 534.32019.

#### 2.1.5. Synthesis of 2-(2-(Isoquinolin-5-yloxy)ethoxy)ethyl (4R,4aS,6aR,9S,11aR,11bS)-4,9,11b-trimethyl-8-oxotetradecahydro-6a,9-methanocyclohepta[a]naphthalene-4-carboxylate(**5**)

Compound **2** (1.4 g, 2.5 mmol) and 5-hydroxyquinoline (0.435 g, 3 mmol) were dissolved in acetonitrile (30 mL). Potassium carbonate (0.691 g, 5 mmol) was added to the solution, and the mixture was heated and refluxed at 80 °C for 24 h. At the end of the reaction, the precipitate was filtered out, and the resulting brown-black oily liquid was concentrated. Compound 5 was then purified using column chromatography and a solvent system consisting of 2:1 petroleum ether and ethyl acetate. Yield: 47%. ^1^H NMR (400 MHz, Chloroform-d) δ 9.21 (d, J = 1.0 Hz, 1H), 8.53 (d, J = 5.8 Hz, 1H), 8.15–7.95 (m, 1H), 7.64–7.40 (m, 2H), 7.03 (dd, J = 7.4, 1.2 Hz, 1H), 4.39–4.16 (m, 4H), 4.05–3.95 (m, 2H), 3.84 (ddd, J = 5.5, 4.0, 1.3 Hz, 2H), 2.59 (dd, J = 18.5, 3.7 Hz, 1H), 2.24–2.14 (m, 1H), 1.90–1.30 (m, 13H), 1.19 (s, 3H), 1.17–1.06 (m, 3H), 1.06–0.94 (m, 4H), 0.86 (td, J = 13.3, 4.4 Hz, 1H), 0.70 (s, 3H). 13C NMR (101 MHz, CDCl3) δ 222.22, 178.02, 153.60, 151.81, 144.25, 130.29, 128.53, 127.38, 118.73, 114.22, 108.73, 77.34, 77.02, 76.70, 69.47, 69.41, 68.12, 63.08, 57.14, 54.67, 54.27, 48.66, 48.40, 43.86, 41.47, 39.75, 39.41, 38.01, 37.89, 37.30, 28.90, 21.61, 20.28, 19.83, 18.91, 13.27. HRMS: Calculated *m*/*z* for C_33_H_43_NO_5_ (M + H): 534.32065, found 534.32092.

### 2.2. Fluorescence Spectral Study

Solutions of probes **4** and **5** (1 × 10^−5^ mol/L) were prepared in ethanol and titrated with ethanol solutions of fumaric acid and maleic acid at gradient concentrations. The Horiba FluoroMax-4 fluorescence spectrophotometer was used to measure the fluorescence during titration in a standard rectangular quartz cuvette (10 × 10 × 45 mm^3^). The corresponding emission values were recorded throughout the titration process.

### 2.3. Detection Limit

The detection limits were determined through fluorescence titration. Fluorescence emission spectra of probes **4** and **5** were recorded five times to calculate the standard deviation of blank measurements. The slope was obtained from the linear relationship between the ratiometric fluorescence intensity (probe **4**: I_488_/I_400_; probe **5**: I_442_/I_364_) and the concentration of maleic acid. The detection limit (LOD) was calculated using the following formula:

LOD = 3σ/k(1)

where σ represents the standard deviation of blank measurements, and k denotes the slope of the calibration curve.

### 2.4. DFT Calculation

Geometry optimization, energy calculations, molecular orbital analysis, and interaction studies were performed using the Gaussian 16 software with the DFT method at the B3LYP/6-31G (d, p) level, in combination with the IEFPCM solvent model and GD3BJ dispersion correction. DFT offers high computational efficiency and a low cost, making it suitable for studying medium-sized molecules [30]. The B3LYP hybrid functional is relatively reliable for non-covalent interaction calculations, while the 6-31G (d, p) basis set provides reasonable geometric optimization accuracy and effectively balances computational cost and precision [31]. The IEFPCM solvent model accounts for solvent effects, making the calculations more representative of real environments [32], whereas the GD3BJ dispersion correction compensates for the underestimation of van der Waals forces in DFT, thereby improving the accuracy of non-covalent interaction calculations [33]. This combination is well-suited for studying the molecular recognition process of fluorescent probes.

### 2.5. Detection of Maleic Acid in Food Additives

Samples for detecting maleic acid in starch-rich foods can be prepared according to the following procedure. First, chop 10 g of starch-rich food and place it in a 500 mL beaker. Add 200 mL of ethanol and stir the mixture for 12 h. Filter the mixture and collect the filtrate. Take 30 mL of the solution and divide it into three portions, adding maleic acid and fumaric acid to two portions, respectively, while the third portion serves as a control. Conduct fluorescence spectroscopy studies on the three solutions.

## 3. Results and Discussion

### 3.1. Fluorescence Sensing Study

**Figure 2:**
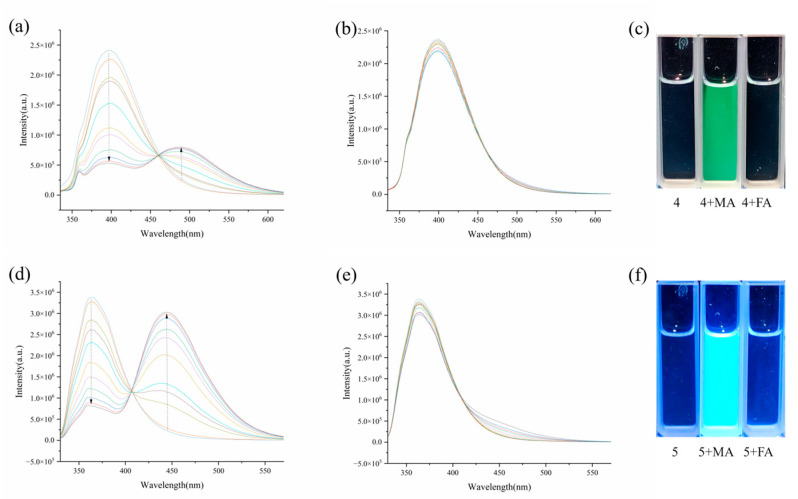
Fluorescence changes of probe **4** at different concentrations of (**a**) maleic acid (0–5 mM) and (**b**) fumaric acid (0–5 mM). (**c**) Fluorescence color changes of probe **4** upon addition of maleic acid and fumaric acid under UV light. Fluorescence changes of probe **5** at different concentrations of (**d**) maleic acid (0–5 mM) and (**e**) fumaric acid (0–5 mM). (**f**) Fluorescence color changes of probe **5** upon addition of maleic acid and fumaric acid under UV light.

To gain a deeper understanding of the interactions between probes **4** and **5** and maleic acid, fluorescence titration experiments were conducted. As shown in Figure 2a, probe **4** exhibits fluorescence emission at 400 nm when excited at 325 nm. Upon gradual addition of maleic acid (0–5 mM) to the ethanol solution of probe **4**, the fluorescence intensity at 400 nm progressively decreases, while a new emission peak appears at 488 nm, with increasing intensity. This results in a typical ratiometric fluorescence signal. In contrast, when fumaric acid is added under the same experimental conditions, no significant fluorescence changes are observed (Figure 2b). The Stokes shift for probe **4** is 88 nm, and after the addition of maleic acid, it emits green fluorescence under UV light (Figure 2c).

Probe **5** exhibits a similar response to maleic acid. As shown in Figure 2d, when maleic acid (0–5 mM) is gradually added to the solution of probe **5**, the fluorescence intensity at 364 nm decreases, while a new emission peak appears at 442 nm. In this case, no significant fluorescence changes occur upon the addition of fumaric acid (Figure 2e). The Stokes shift for probe **5** is 78 nm, and following the addition of maleic acid, it emits pale blue fluorescence under UV light (Figure 2f).

These results indicate that probes **4** and **5** interact with maleic acid, producing distinct spectral responses that effectively differentiate between maleic acid and fumaric acid. It should be mentioned here that the detection limit for maleic acid was determined from the fluorescence titration experiment using Equation (1), and it was found to be 4.14 × 10^−6^ M (probe **4**) and 1.88 × 10^−6^ M (probe **5**) (Figure S10, Supplementary Materials).

**Figure 3:**
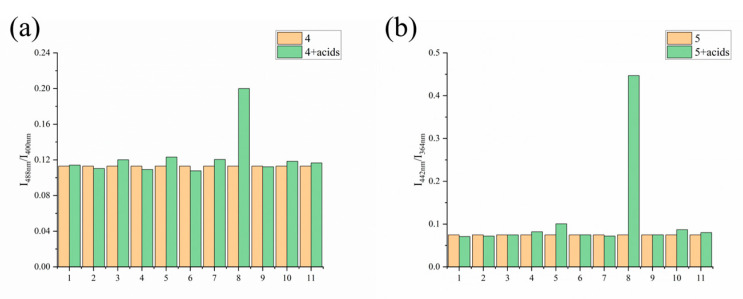
Anti-carboxylic acid interference effect of probe **4** (**a**) and probe **5** (**b**). (1: lauric acid, 2: benzoic acid, 3: acetic acid, 4: malic acid, 5: malonic acid, 6: caproic acid, 7: succinic acid, 8: maleic acid, 9: fumaric acid, 10: tartaric acid and 11: citric acid) (the concentration of carboxylic acids added is set to 0.1 mM).

To examine the fluorescence responses of probes **4** and **5** to different carboxylic acids, we recorded the fluorescence changes of probes **4** and **5** in the absence and presence of various carboxylic acids. In the presence of all other carboxylic acids (except maleic acid), the fluorescence spectra of probes **4** and **5** were almost unchanged (Figure 3). The experimental results support the specific recognition of maleic acid by probes **4** and **5**. Additionally, we tested the effects of temperature and pH on the fluorescence performance of probes **4** and **5** and found that they remained stable within the temperature range of 0–60 °C and the pH range of 4–11 (Figures S11 and S12, Supplementary Materials).

### 3.2. ^1^H NMR Study

**Figure 4:**
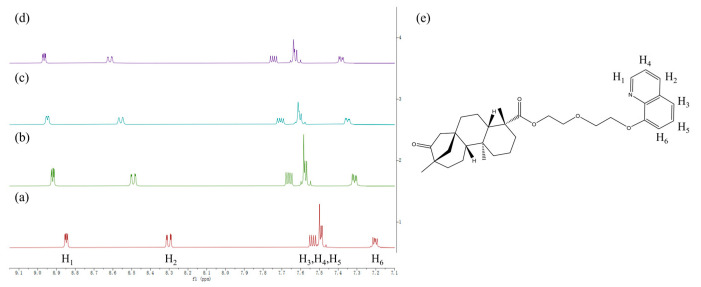
(**a**) Partial ^1^H NMR of **4**, (**b**) partial ^1^H NMR of **4** with 0.5 equivalent of maleic acid, (**c**) partial ^1^H NMR of **4** with 1 equivalent of maleic acid, (**d**) partial ^1^H NMR of **4** with 2 equivalents of maleic acid in DMSO-d_6_ and (**e**) protons involved in the ^1^H NMR.

To further investigate the exact mechanism behind this fluorescence change, we conducted ^1^H NMR tests in DMSO-d6 by mixing the host and guest at ratios of 1:0.5, 1:1, and 1:2. The results are shown in Figure 4. Upon mixing with maleic acid, the protons of the quinoline moiety in probe **4** exhibited downfield shifts (ΔδH_1_ = 0.12 ppm, ΔδH_2_ = 0.32 ppm, ΔδH_3_ = 0.20 ppm, ΔδH_4_ = 0.14 ppm, ΔδH_5_ = 0.14 ppm, ΔδH_6_ = 0.18 ppm). In contrast, when probe **4** was mixed with fumaric acid, the changes in chemical shifts were negligible (Figure S13, Supplementary Materials).

**Figure 5:**
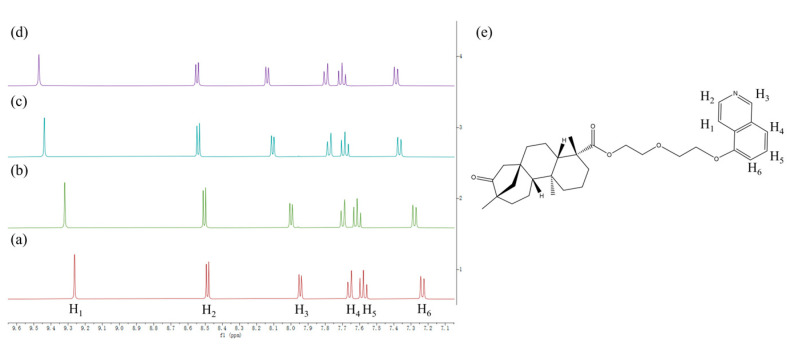
(**a**) Partial ^1^H NMR of **5**, (**b**) partial ^1^H NMR of **5** with 0.5 equivalent of maleic acid, (**c**) partial ^1^H NMR of **5** with 1 equivalent of maleic acid, (**d**) partial ^1^H NMR of **5** with 2 equivalents of maleic acid in DMSO-d_6_ and (**e**) protons involved in the ^1^H NMR.

Similarly, as shown in Figure 5, when probe **5** is mixed with maleic acid, the chemical shifts of protons on the isoquinoline group shift downfield (ΔδH_1_ = 0.21 ppm, ΔδH_2_ = 0.06 ppm, ΔδH_3_ = 0.20 ppm, ΔδH_4_ = 0.14 ppm, ΔδH_5_ = 0.12 ppm, ΔδH_6_ = 0.16 ppm). However, no significant changes are observed when probe **5** is mixed with fumaric acid (Figure S14, Supplementary Materials).

The observed downfield shifts of quinoline and isoquinoline protons upon interaction with maleic acid in DMSO-d_6_ indicate hydrogen bonding or other non-covalent interactions. However, the relatively small chemical shift changes (~0.15 ppm) suggest that the solvent may competitively interact with the probe or the guest molecule, thereby attenuating the binding-induced perturbation. In contrast, the larger chemical shift changes observed in CDCl_3_ (Figures S15 and S16, Supplementary Materials) imply a stronger interaction between the probe and maleic acid in a less polar environment, where solvent competition is minimized, leading to more pronounced deshielding effects.

### 3.3. Density Functional Theory (DFT) Studies

Combined with the experimental evidence, the possible 3D structures of probes **4** and **5** with maleic acid and fumaric acid complexes in ethanol were calculated by using the density functional theory (DFT) method at the B3LYP/6-31G(d, p) level, employing the IEFPCM solvent model and GD3BJ dispersion correction.

**Figure 6:**
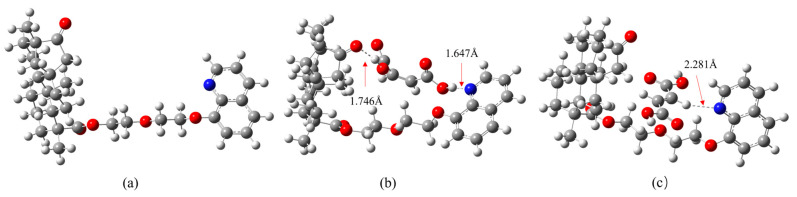
3D structure of **4** (**a**), **4-MA** complex (**b**), and **4-FA** complex (**c**) calculated by B3LYP/6-31G (d, p).

Figure 6b illustrates the formation of intermolecular hydrogen bonds in the probe **4**-maleic acid complex, as highlighted by the red arrow markings. Compared to fumaric acid, the C=O and quinoline-N of probe **4** form stronger hydrogen bonds with the carboxyl group of maleic acid. The carboxyl group of maleic acid is positioned closer to the quinoline group, and its electron-withdrawing effect reduces the electron cloud density of the quinoline proton, causing a downfield chemical shift.

The C=O group of probe **4** forms a hydrogen bond with the carboxyl group of maleic acid, which is consistent with the fluorescence quenching observed due to the photoelectron transfer (PET) process. As the concentration of maleic acid increases, the π-π stacking interactions between the fluorophore quinoline groups are enhanced, resulting in an Excimer effect. This leads to the emergence and progressive enhancement of the emission peak associated with exciton formation, which corresponds to the appearance and intensification of the new fluorescence emission peaks.

**Figure 7:**
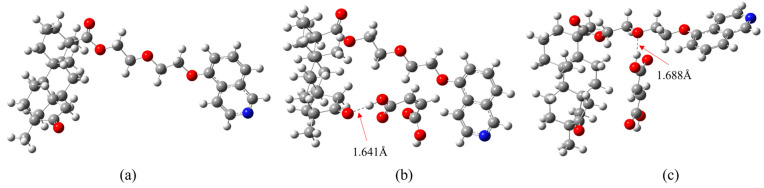
3D structure of **5** (**a**), **5-MA** complex (**b**) and **5-FA** complex (**c**) calculated by B3LYP/6-31G (d, p).

Figure 7b reveals the formation of intermolecular hydrogen bonds in the probe **5**-maleic acid complex, as indicated by the red arrow markers. Similarly, the complexation of probe **5** with maleic acid resulted in a chemical shift of the isoquinoline group proton to a low-field shift and a ratiometric change in fluorescence.

### 3.4. Identification of Maleic Acid in Starchy Food

Because maleic anhydride can combine with edible starch to produce modified starch with a low gelatinization temperature, high viscosity, good stability, and strong adhesiveness, unscrupulous manufacturers add maleic anhydride to food-grade modified starch to enhance the elasticity, viscosity, and glossiness of food. When exposed to water, maleic anhydride converts into maleic acid, which can be harmful to the human body. Therefore, it is crucial to develop a method to detect maleic acid and maleic anhydride in edible starch. We anticipate that probes **4** and **5** will offer a promising approach for the detection of maleic acid in starchy food samples.

**Figure 8:**
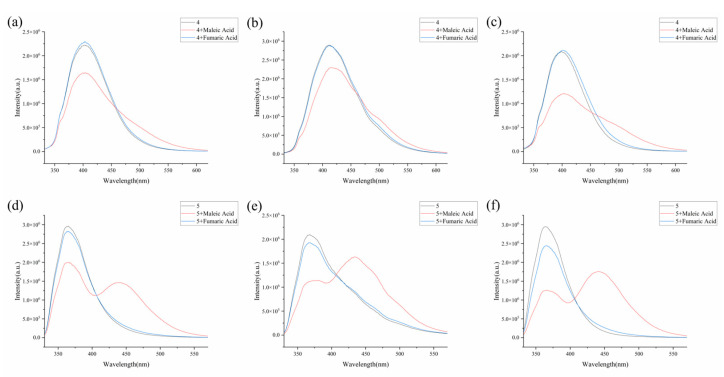
Determination of maleic acid in starch-rich foods: probe **4**: (**a**) potato, (**b**) sweet potato, and (**c**) corn; probe **5**: (**d**) potato, (**e**) sweet potato, and (**f**) corn.

In our experiment, we prepared the samples through a simple liquid extraction of starch-rich foods, and divided them into three parts. Maleic acid and fumaric acid were added to two of the parts. Using fluorescence spectroscopy, probes **4** and **5** exhibited distinct responses to maleic acid versus fumaric acid in each starch-rich food sample (Figure 8). These findings suggest that probes **4** and **5** are well-suited for the detection of maleic acid in starch-rich food samples.

## 4. Conclusions

In summary, we synthesized novel ratiometric fluorescent probes (**4** and **5**) that can effectively distinguish maleic acid from its trans isomer, fumaric acid, based on their distinct fluorescence responses. The detection limits for probes **4** and **5** are 4.14 × 10^−6^ M and 1.88 × 10^−6^ M, respectively. The formation of hydrogen bonds between probes 4 and 5 and maleic acid was confirmed through ^1^H NMR and DFT calculations. Finally, the practical application of probes **4** and **5** was demonstrated by qualitatively detecting maleic acid in starch-rich foods such as corn, potato, and sweet potato.

## Data Availability

The original contributions presented in the study are included in the article/Supplementary Materials. Further inquiries can be directed to the corresponding authors.

## Supplementary Materials

The following supporting information can be downloaded at: https://www.mdpi.com/article/10.3390/foods14091541/s1, Figure S1: ^1^H NMR spectra of **1**; Figure S2: ^1^H NMR spectra of **2**; Figure S3: ^1^H NMR spectra of **3**; Figure S4: ^1^H NMR spectra of **4**; Figure S5: ^13^C NMR spectra of **4**; Figure S6: ^1^H NMR spectra of **5**; Figure S7: ^13^C NMR spectra of **5**; Figure S8: HRMS spectra of **4**; Figure S9: HRMS spectra of **5**; Figure S10: Linear relationship diagram of Probe **4**(a) and Probe **5**(b); Figure S11: Effect of temperature on the performance of probes **4**(a) and **5**(b); Figure S12: Effect of pH on the performance of probes **4**(a) and **5**(b); Figure S13: (a) Partial ^1^H NMR of **4**, (b) Partial ^1^H NMR of **4** with 0.5 equivalent of fumaric acid, (c) Partial ^1^H NMR of **4** with 1 equivalent of fumaric acid and (d) Partial ^1^H NMR of **4** with 2 equivalents of fumaric acid in DMSO-d_6_; Figure S14: (a) Partial ^1^H NMR of **5**, (b) Partial ^1^H NMR of **5** with 0.5 equivalent of fumaric acid, (c) Partial ^1^H NMR of **5** with 1 equivalent of fumaric acid and (d) Partial ^1^H NMR of **5** with 2 equivalents of fumaric acid in DMSO-d_6_; Figure S15: (a) Partial ^1^H NMR of **4**, (b) Partial ^1^H NMR of **4** with 1 equivalent of maleic acid, (c) Partial ^1^H NMR of **4** with 2 equivalents of maleic acid in CDCl_3_; Figure S16: (a) Partial ^1^H NMR of **5**, (b) Partial ^1^H NMR of **5** with 1 equivalent of maleic acid, (c) Partial ^1^H NMR of **5** with 2 equivalents of maleic acid in CDCl_3_; Table S1: HOMO and LUMO Energy Gap of **4**, **4-MA** and **4-FA** calculated on Gaussian 16; Table S2: Cartesian coordinates of the optimized geometry of **4** in the solvent phase; Table S3: Cartesian coordinates of the optimized geometry of **4-MA** in the solvent phase; Table S4: Cartesian coordinates of the optimized geometry of **4-FA** in the solvent phase; Table S5: HOMO and LUMO Energy Gap of **5**, **5-MA** and **5-FA**; Table S6: Cartesian coordinates of the optimized geometry of **5** in the solvent phase; Table S7: Cartesian coordinates of the optimized geometry of **5-MA** in the solvent phase; Table S8: Cartesian coordinates of the optimized geometry of **5-FA** in the solvent phase. Relevant references used in the supplementary section include [34].
