# Supplementary material for: Ratiometric Fluorescent Probes Based on Isosteviol with Identification of Maleic Acid in Starchy Foods
Source: Foods. 2025 Apr 28;14(9):1541. doi: 10.3390/foods14091541 (PMC12071534; doi:10.3390/foods14091541)
Supplement: Supplementary file 1 [file foods-14-01541-s001.zip › foods-3507417-supplementary.pdf]

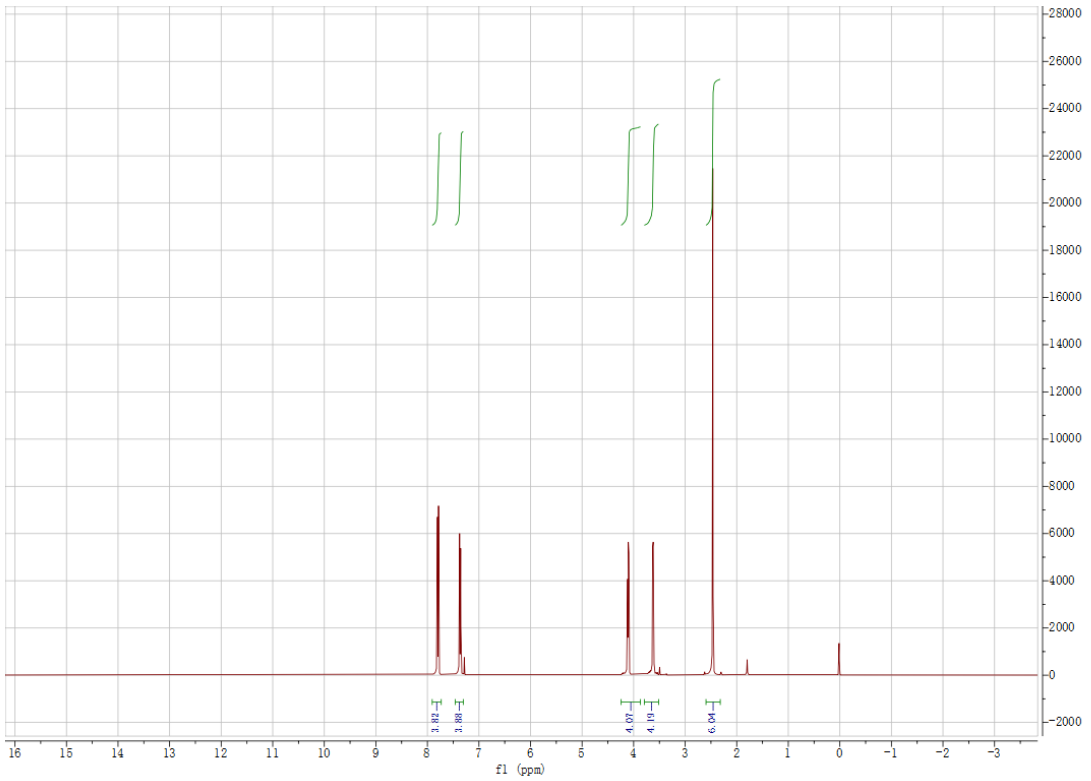

Figure S1. ^{1}H NMR spectra of 1.

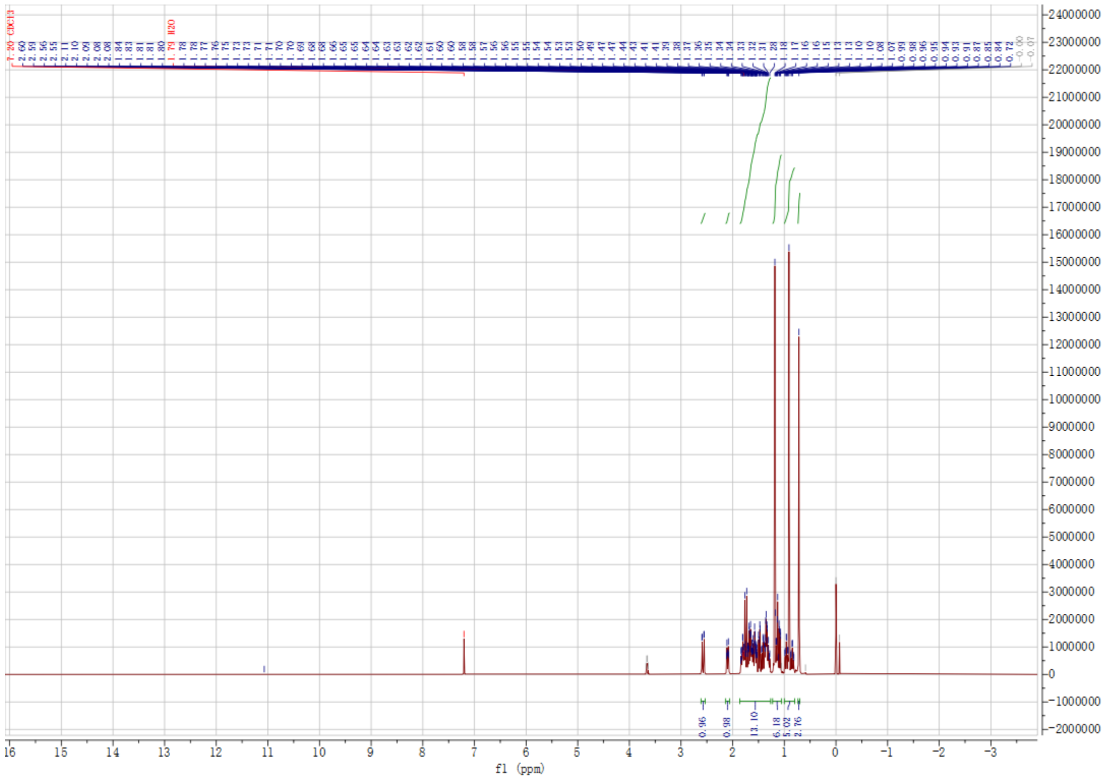

Figure S2. ^{1}H NMR spectra of 2.

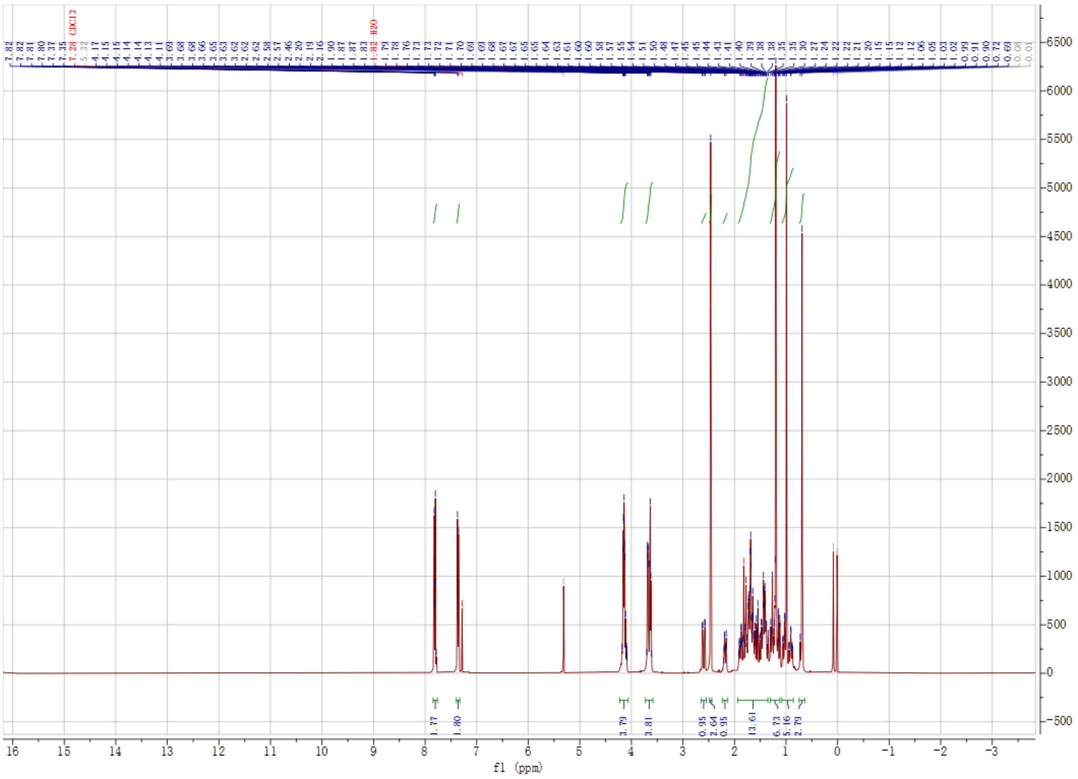

Figure S3. ^{1}H NMR spectra of 3.

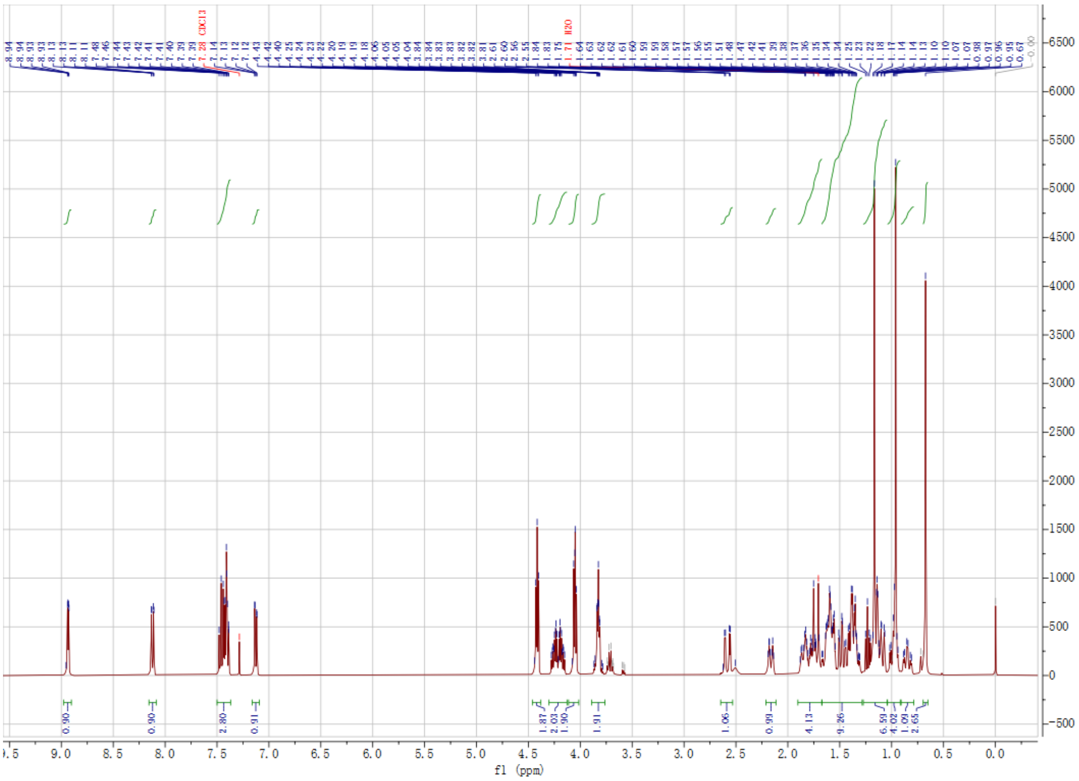

Figure S4. ^{1}H NMR spectra of 4.

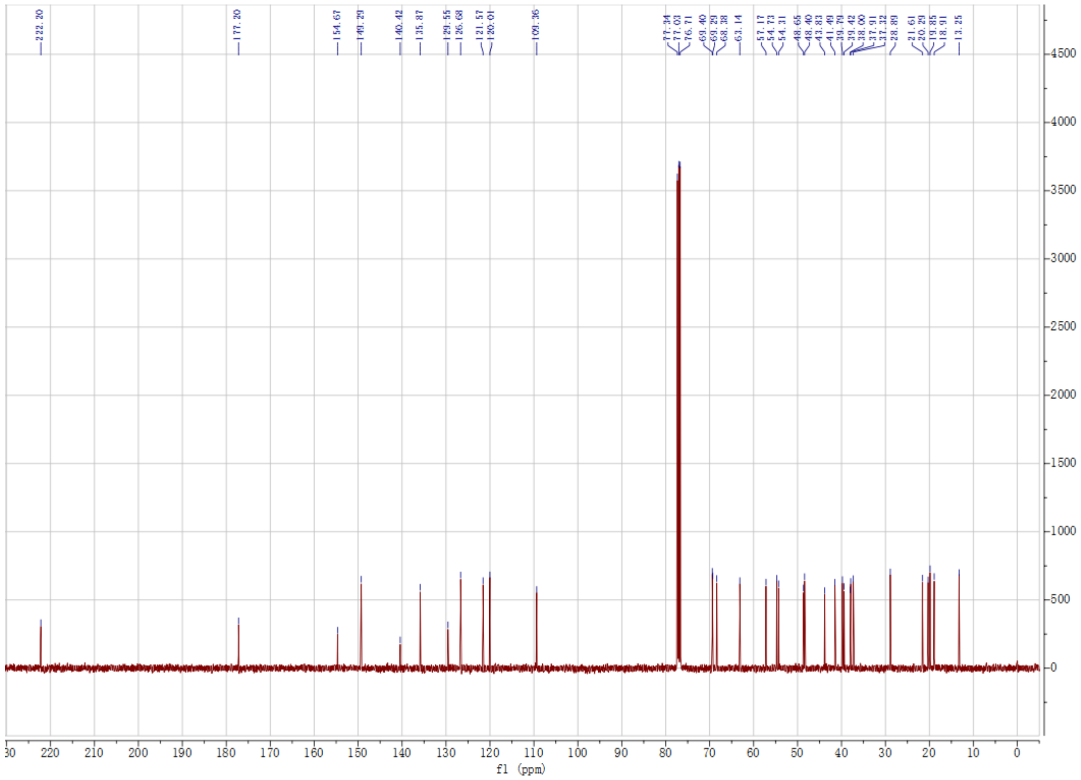

Figure S5. ^{13}C NMR spectra of 4.

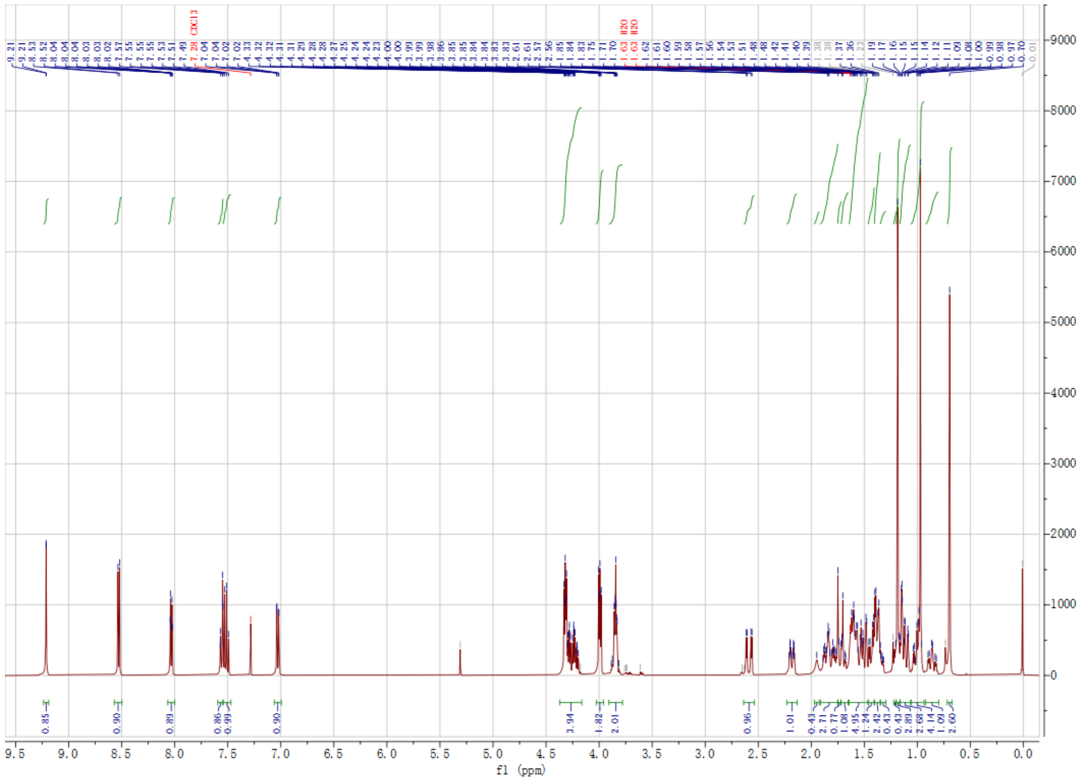

Figure S6. ^{1}H NMR spectra of 5.

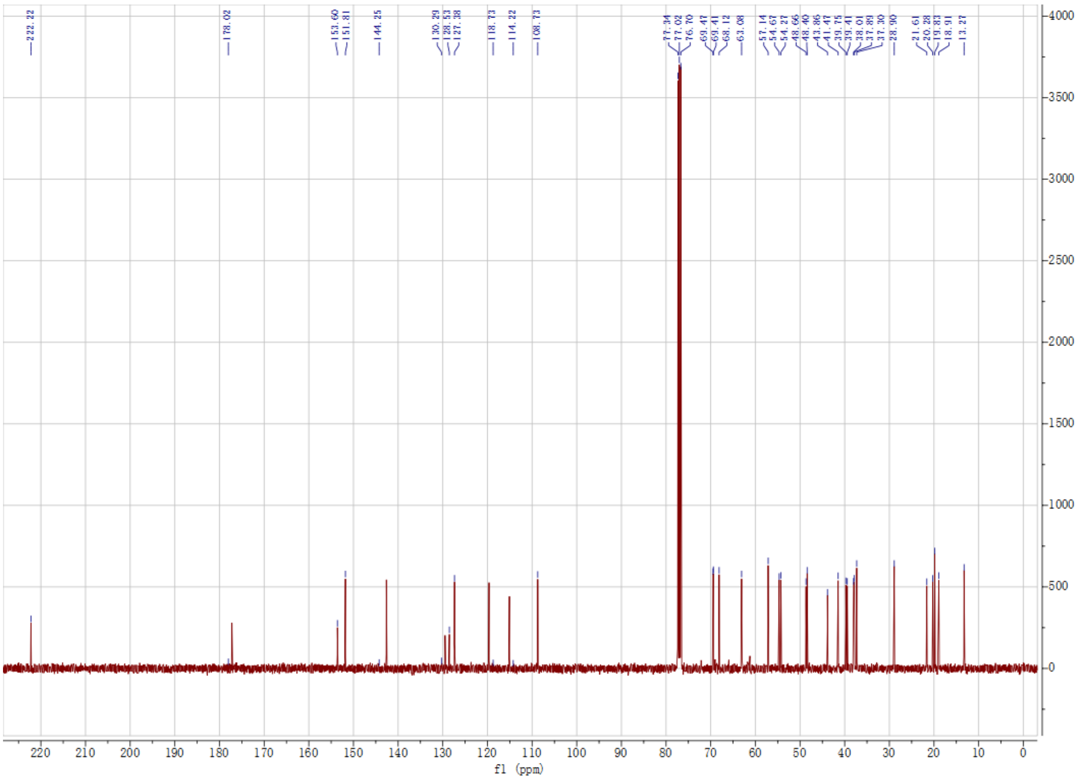

Figure S7. ^{13}CNMR spectra of 5.

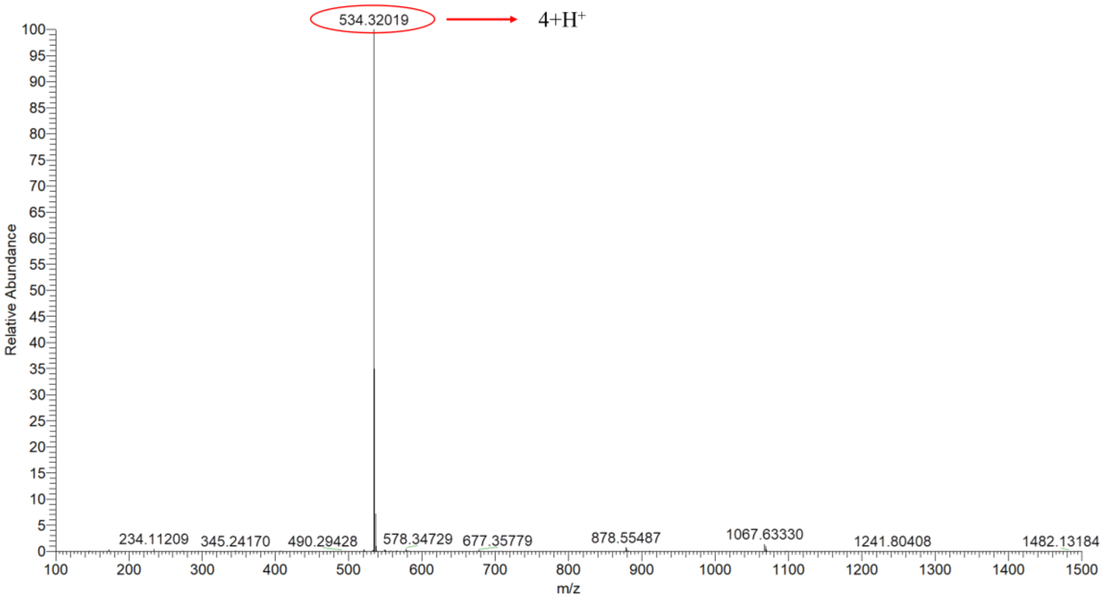

Figure S8. HRMS spectra of 4.

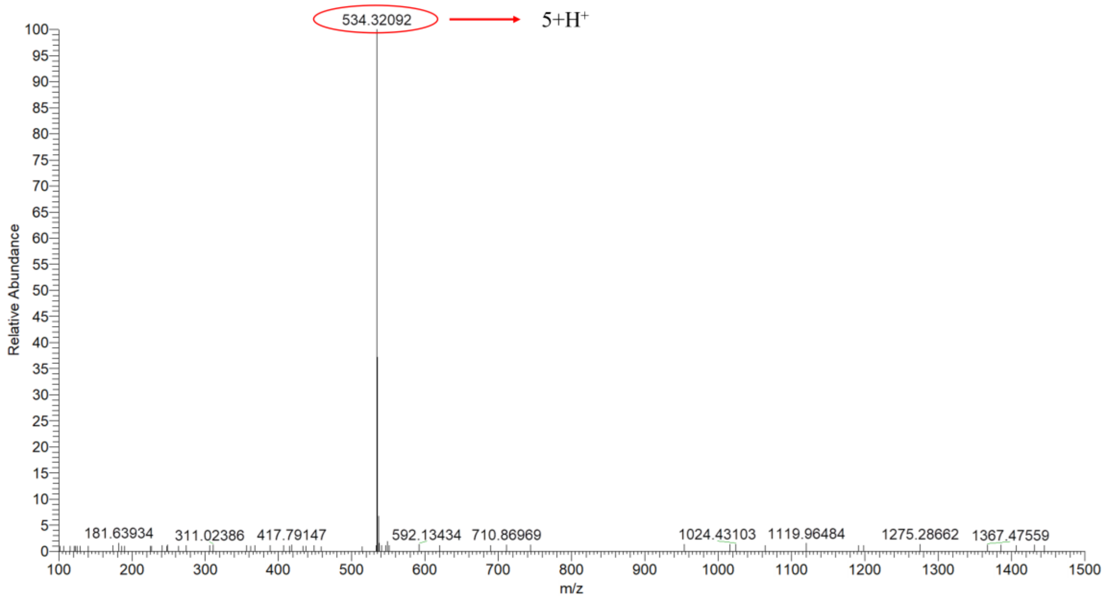

Figure S9. HRMS spectra of 5.

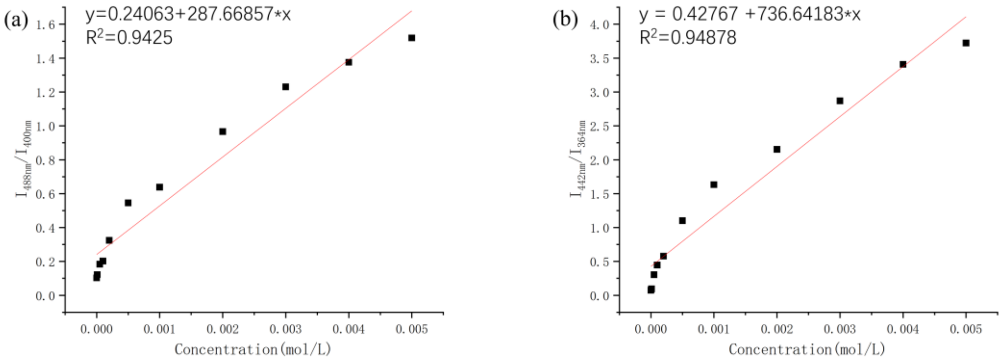

Figure S10. Linear relationship diagram of Probe 4(a) and Probe 5(b).

Although no official limit exists for maleic acid, the regulatory threshold for its structurally related compound, fumaric acid, is 3g/kg(http://www.nhc.gov.cn/wjw/aqbz/201106/a2cedb1c45894acdba5790ca164883aa.shtml). Assuming that the density of food is approximately equal to that of water (1kg ≈ 1L), the maximum permitted level (3g/kg) corresponds to approximately 25.84 mM. The detection limit of our ratiometric fluorescence method was determined to be 4.14 μM (probe 4) and 1.88μM (probe 5), which is significantly lower than the regulatory maximum usage level of 25.84 mM. This indicates that our method is highly sensitive and capable of detecting trace amounts of the target compound, well below safety thresholds.

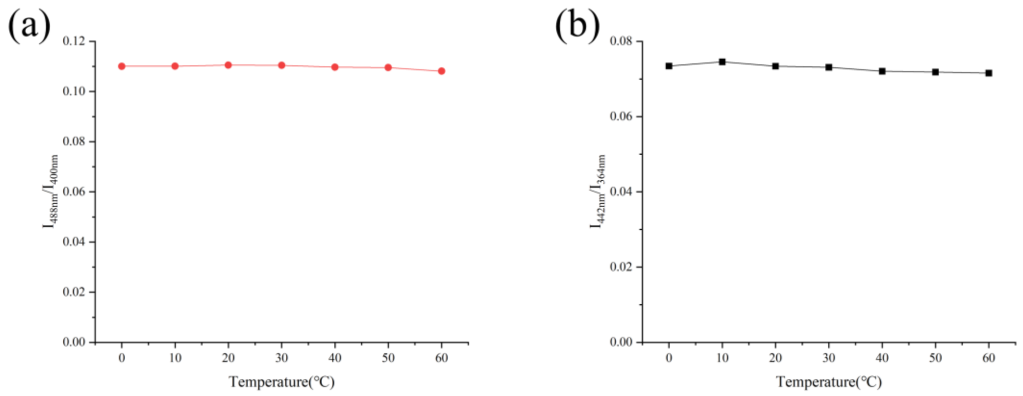

Figure S11. Effect of temperature on the performance of probes 4(a) and 5(b).

To assess the temperature stability of the probes, fluorescence measurements were conducted at different temperatures ranging from 0 to 60 °C. The results showed no significant variation in fluorescence intensity, indicating the robustness of the probes under typical experimental conditions.

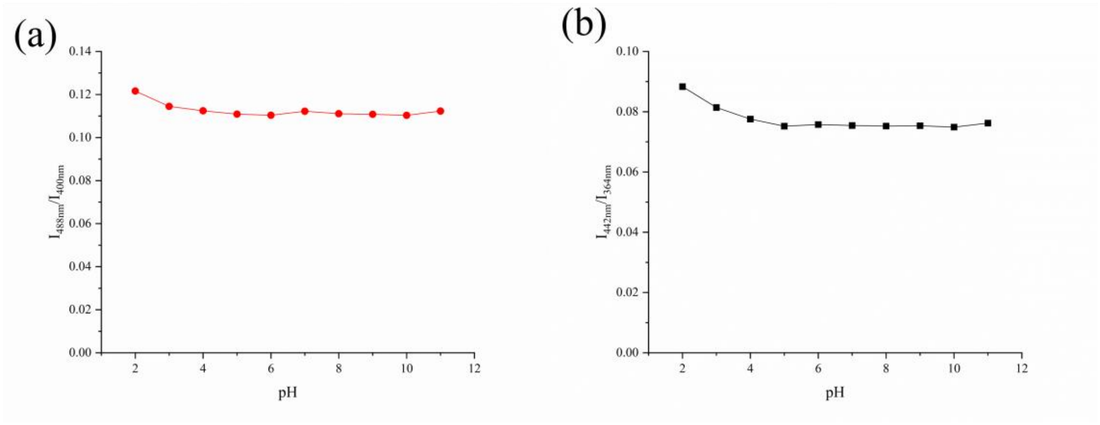

Figure S12. Effect of pH on the performance of probes 4(a) and 5(b).

To evaluate the pH stability of the probes, its fluorescence response was examined in solutions with pH values ranging from 2 to 11. Although extreme acidic conditions (pH = 2) were found to slightly affect the fluorescence response of the probe, its overall performance remained stable, indicating that pH fluctuations in real samples would not significantly impact its detection performance. In the future, we will consider using appropriate buffer systems (such as HEPES or PBS) to maintain pH stability and minimize the influence of environmental acidity on the probe.

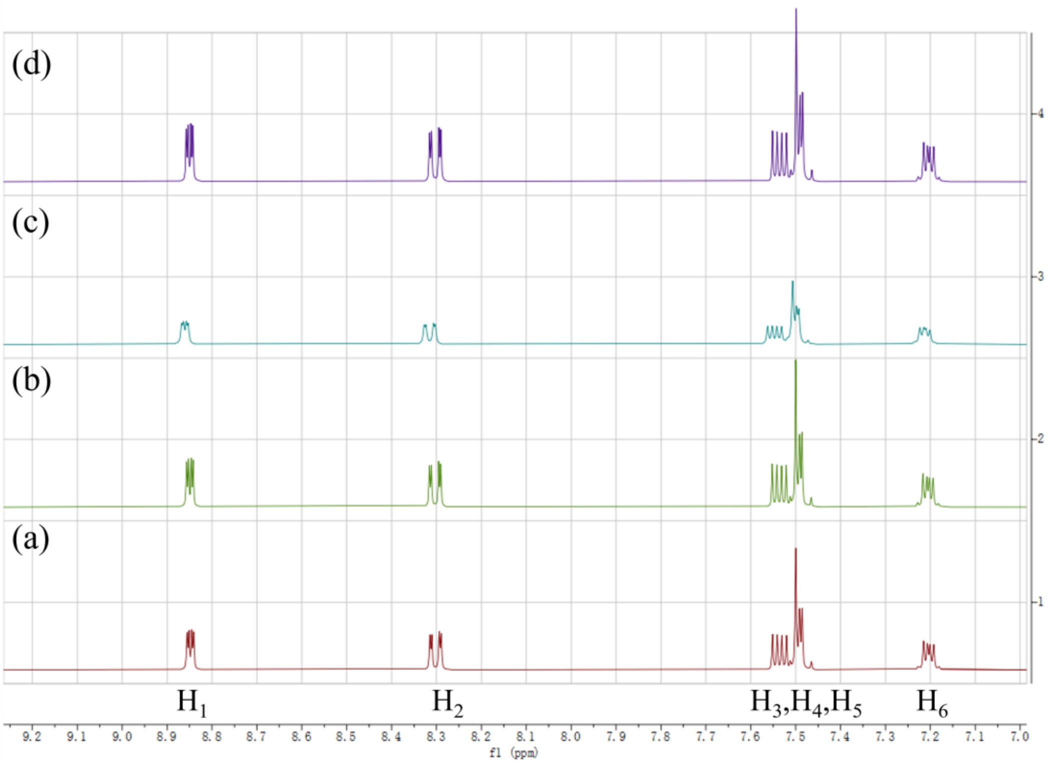

Figure S13. (a) Partial ^{1}H NMR of 4, (b) Partial ^{1}H NMR of 4 with 0.5 equivalent of fumaric acid, (c) Partial ^{1}H NMR of 4 with 1 equivalent of fumaric acid and (d) Partial ^{1}H NMR of 4 with 2 equivalents of fumaric acid in DMSO-d_6.

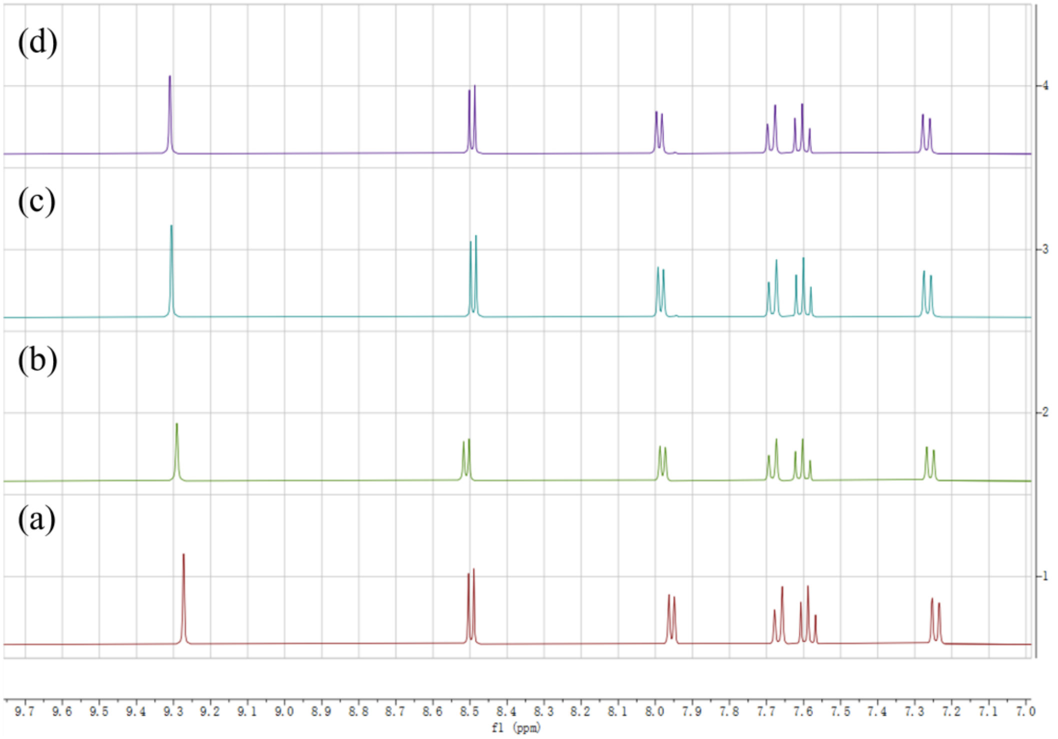

Figure S14. (a) Partial ^{1}H NMR of 5, (b) Partial ^{1}H NMR of 5 with 0.5 equivalent of fumaric acid, (c) Partial ^{1}H NMR of 5 with 1 equivalent of fumaric acid and (d) Partial ^{1}H NMR of 5 with 2 equivalents of fumaric acid in DMSO-d_6.

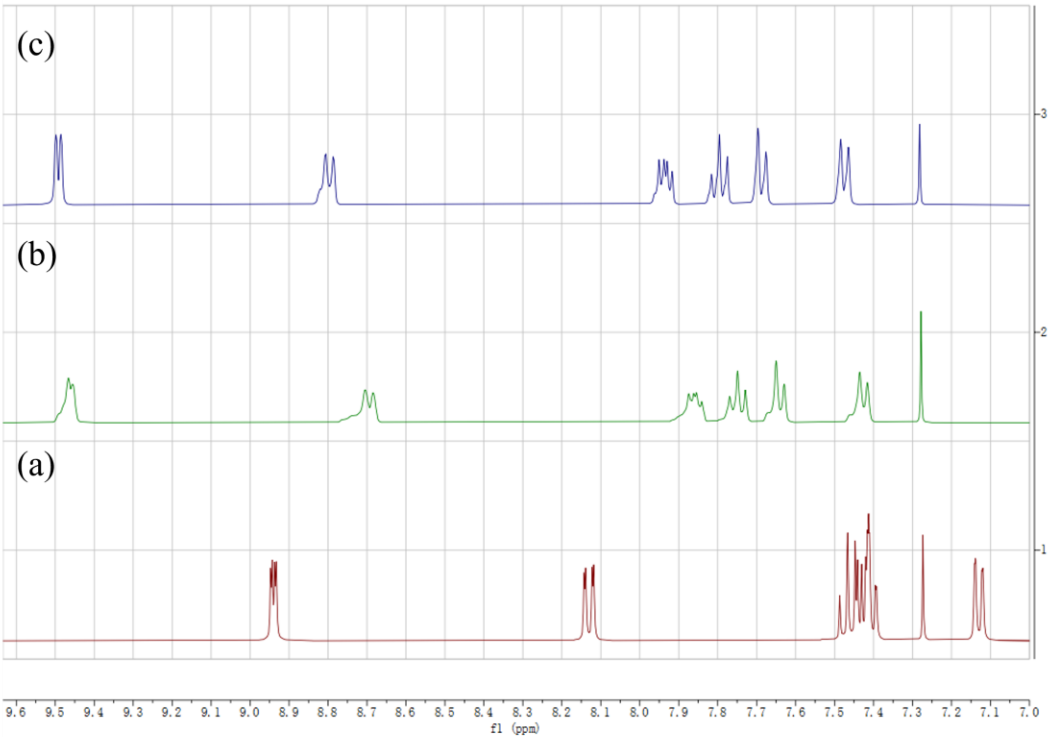

Figure S15. (a) Partial ^{1}H NMR of 4, (b) Partial ^{1}H NMR of 4 with 1 equivalent of maleic acid, (c) Partial ^{1}H NMR of 4 with 2 equivalents of maleic acid in $CDCl_3$.

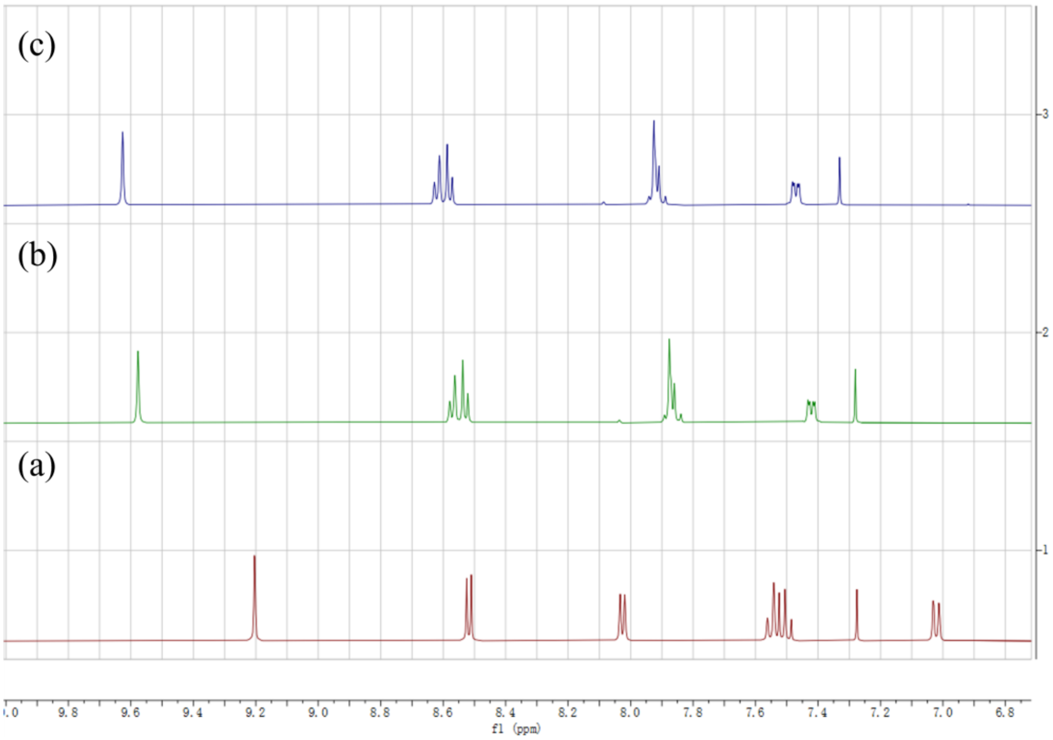

Figure S16. (a) Partial ^{1}H NMR of 5, (b) Partial ^{1}H NMR of 5 with 1 equivalent of maleic acid, (c) Partial ^{1}H NMR of 5 with 2 equivalents of maleic acid in $CDCl_3$.

The data in Tables S1 to S8 were obtained using the Gaussian 16 software through DFT calculations at the B3LYP/6-31G(d,p) level, employing the IEFPCM solvent model and GD3BJ dispersion

correction[1].

Table S1. HOMO and LUMO Energy Gap of 4, 4-MA and 4-FA calculated on Gaussian 16.

	4	4-MA	4-FA
HOMO	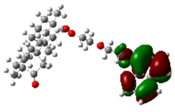	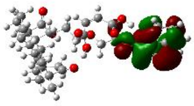	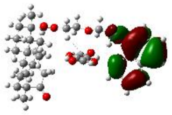
LUMO	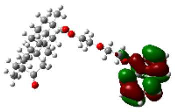	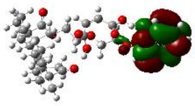	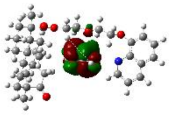
E_h	-6.182153422eV	-6.23929732eV	-6.196847567eV
E_l	-1.526286304eV	-1.788331894eV	-2.44684729eV
ΔE	4.655867118eV	4.450965427eV	3.750000278eV

Table S2. Cartesian coordinates of the optimized geometry of 4 in the solvent phase.

	**4**		
N	-6.56847542	1.62087587	0.79445738
C	-6.95090075	2.41426042	1.77811485
C	-8.29552628	2.57854187	2.19086161
C	-9.27645812	1.87703776	1.53140664
C	-8.92223545	1.01069883	0.46441101
C	-9.88177012	0.27019394	-0.27512793
C	-9.47734519	-0.5407792	-1.31094074
C	-8.10636499	-0.65553228	-1.64082627
C	-7.14774654	0.04572488	-0.93923368
C	-7.53104	0.9122253	0.13499079
O	-5.83676552	-0.04733504	-1.34048726
C	2.99526863	-0.2745816	1.58210321
C	6.65963948	4.4325341	-0.92489977
O	4.02390289	4.56822564	0.40148986
C	1.70705439	-2.93762051	0.53350701
O	0.81332425	-2.25366003	-0.21456314
O	1.39074739	-3.47451842	1.57944973
C	-0.53142079	-2.18774049	0.3010468
C	-1.35621458	-1.43849549	-0.73239905
O	-2.68090956	-1.38359866	-0.23324277

C	-3.57356283	-0.70551861	-1.10200776
C	-4.9425526	-0.72122638	-0.44069442
C	2.90280816	-3.86213579	-1.41390361
C	4.50586507	1.24690073	-0.98539905
C	5.0971106	0.41411262	0.20150309
C	5.65116999	2.12344947	-1.56451718
C	3.93999691	0.31853526	-2.06779007
C	3.52178545	2.3566821	-0.51922782
C	4.20294995	-0.76741289	0.75141085
C	5.63381912	1.36063895	1.30114434
C	5.85988967	3.20662953	-0.49335689
C	3.01335978	-0.77280373	-1.52683478
C	4.40731943	3.53833747	-0.12300194
C	3.75134701	-1.6301472	-0.48106067
C	5.10630012	-1.64731925	1.66146849
C	6.49248694	2.51413533	0.74681829
C	3.09178544	-3.01639166	-0.12605172
C	4.49044371	-2.99830848	2.04260971
C	4.05190018	-3.79011589	0.80898221
H	5.97323351	-0.09297757	-0.23150336
H	4.69032171	-1.94105392	-0.9670259
H	-6.16364138	2.96839936	2.28715438
H	-8.53129406	3.24956767	3.00984517
H	-10.32183238	1.97587086	1.81062654
H	-10.93213764	0.35949188	-0.01393729
H	-10.20823422	-1.10499197	-1.88174166
H	-7.78617804	-1.29649223	-2.45577175
H	2.49935416	-1.10370754	2.09073738
H	3.3114419	0.4208248	2.36428763
H	2.23770941	0.22752046	0.97739701
H	7.70550492	4.16861838	-1.11300071
H	6.2501625	4.86818331	-1.8424825
H	6.63437047	5.20103424	-0.14628509
H	-0.92002594	-3.19762098	0.45602075
H	-0.53586078	-1.66531067	1.26170475
H	-0.94949387	-0.42711573	-0.88421007
H	-1.3189334	-1.96355242	-1.69900472
H	-3.62204861	-1.20165563	-2.08326762
H	-3.25058293	0.33369758	-1.26654619
H	-4.90782284	-0.20441966	0.52197436
H	-5.2760613	-1.75504242	-0.28393721
H	2.14964198	-3.44352784	-2.08269095
H	3.8518839	-3.92814299	-1.95534972
H	2.59312136	-4.879892	-1.15403488

H	6.55996085	1.54477633	-1.76322916
H	5.33017451	2.57454413	-2.51338089
H	3.42178741	0.91833666	-2.82711052
H	4.78459269	-0.16378391	-2.58097033
H	2.83671636	2.1014604	0.29059448
H	2.90326044	2.68525331	-1.36631429
H	6.22811649	0.80121639	2.02915338
H	4.79791503	1.78985291	1.86543225
H	2.1092479	-0.32806871	-1.1022226
H	2.67936878	-1.39563933	-2.36142899
H	6.05344059	-1.83170928	1.1347876
H	5.36130183	-1.09879676	2.57480943
H	6.66215037	3.25559193	1.53637062
H	7.47779439	2.13888724	0.44126835
H	5.22746856	-3.58266938	2.60676221
H	3.63517616	-2.86030457	2.71106855
H	4.93857669	-4.05062813	0.21605886
H	3.58231439	-4.73211795	1.10838635

Table S3. Cartesian coordinates of the optimized geometry of 4-MA in the solvent phase.

	**4-MA**		
N	6.20238488	0.53721367	-0.94272571
C	7.05925699	1.09650394	-1.77862346
C	8.43779394	1.23429103	-1.50412417
C	8.92601568	0.73574305	-0.31984101
C	8.0442634	0.11868881	0.60377759
C	8.48278375	-0.43641481	1.83216116
C	7.57382604	-1.0199293	2.68532361
C	6.1981866	-1.05379972	2.36416905
C	5.73858319	-0.52174061	1.17611856
C	6.65957852	0.05839908	0.25198524
O	4.40951878	-0.45119168	0.86894733
C	-2.88945087	-0.43080446	-1.12554257
C	-3.64435747	5.28645683	1.47751506
O	-1.3069358	3.73359905	0.43910407
C	-3.11975999	-3.28512549	-0.34259572
O	-2.15495602	-2.96352834	0.54558365
O	-2.88940054	-3.90974488	-1.36073185
C	-0.7834275	-3.20042773	0.17376416
C	0.02946671	-2.13701435	0.89455237
O	1.39735261	-2.37247826	0.61979166
C	2.21453248	-1.30695079	1.08073369
C	3.65995331	-1.67455109	0.79193698
C	-4.9084997	-3.78504356	1.26364306

C	-3.91675853	1.46612085	1.44362629
C	-4.55391286	1.16196019	0.05154093
C	-4.42354396	2.84557766	1.92905463
C	-4.24530746	0.35390097	2.43436889
C	-2.40129584	1.7576515	1.35890673
C	-4.30071999	-0.28362592	-0.52251381
C	-4.21521134	2.279261	-0.96221175
C	-3.69138146	3.83775108	1.00859965
C	-3.82072077	-1.01334058	1.90695695
C	-2.31188759	3.19113635	0.88849491
C	-4.57203231	-1.33322309	0.60653522
C	-5.33614892	-0.53698075	-1.64896942
C	-4.34320709	3.70209985	-0.39494755
C	-4.50440076	-2.82205106	0.12107849
C	-5.34293599	-1.98065016	-2.16385545
C	-5.5153213	-2.99615081	-1.03280914
H	-5.63761559	1.2075679	0.23385459
H	-5.63791823	-1.21088211	0.85508807
H	6.64982996	1.45157076	-2.72100834
H	9.08603909	1.71226622	-2.22910105
H	9.98208291	0.80424205	-0.07715148
H	9.53729822	-0.39177597	2.08404495
H	7.9045578	-1.44464518	3.6272701
H	5.48884757	-1.48097871	3.06480109
H	-2.78998248	-1.35928236	-1.68645261
H	-2.67894611	0.37285145	-1.83150063
H	-2.10824654	-0.43168138	-0.36789802
H	-4.64885661	5.72030107	1.47697416
H	-3.24248281	5.35832923	2.49305097
H	-3.0109112	5.88340038	0.81516984
H	-0.48771095	-4.20605444	0.48601284
H	-0.67169055	-3.1205578	-0.90891385
H	-0.26559368	-1.14064534	0.53677645
H	-0.16906317	-2.17568923	1.97629578
H	2.06795332	-1.13825387	2.15790657
H	1.96148737	-0.37369469	0.55987081
H	3.74374803	-2.07940722	-0.22119481
H	4.03559476	-2.41064225	1.50796558
H	-4.16175144	-3.82131339	2.05811142
H	-5.86180191	-3.46569664	1.69558969
H	-5.03510249	-4.7985519	0.87098184
H	-5.51131892	2.94361372	1.86311856
H	-4.13401139	2.99761141	2.97644638
H	-3.75778879	0.56628735	3.39378813

H	-5.32762716	0.35402161	2.6232253
H	-1.79445255	1.09390684	0.75085163
H	-1.97571486	1.73657747	2.3729775
H	-4.85013355	2.18929112	-1.8475249
H	-3.18816421	2.16186237	-1.31966155
H	-2.74055593	-1.03296827	1.75161619
H	-4.03503564	-1.77530294	2.66041747
H	-6.33392496	-0.29421777	-1.25931867
H	-5.15246616	0.14361349	-2.48675278
H	-3.87696956	4.4116178	-1.08681202
H	-5.39596178	3.98929737	-0.28873356
H	-6.15729189	-2.10157236	-2.88719661
H	-4.41927549	-2.19817849	-2.70854025
H	-6.51687884	-2.88614266	-0.59845108
H	-5.44518939	-4.01464764	-1.42459075
C	1.64529088	-0.32683022	-2.36934888
C	0.3986036	0.12583431	-2.20838301
C	-0.02934417	1.4586184	-1.68139576
O	-0.67761634	2.22746569	-2.37179943
O	0.25325669	1.66518855	-0.39529284
C	2.86863817	0.43590861	-1.99779468
O	2.87742831	1.62531884	-1.7133805
O	3.94183577	-0.34728053	-2.01692465
H	1.80582287	-1.31270981	-2.79263873
H	-0.43190561	-0.48704134	-2.54706507
H	-0.1762632	2.51972836	-0.10581185
H	4.74189545	0.1077986	-1.57050797

Table S4. Cartesian coordinates of the optimized geometry of 4-FA in the solvent phase.

	**4-FA**		
N	5.08164089	1.13547055	-0.06846858
C	4.93659304	2.33600083	-0.59702564
C	5.99416713	3.08672416	-1.16183598
C	7.2566427	2.54409334	-1.16392522
C	7.46740304	1.25601481	-0.60940221
C	8.74576955	0.64218509	-0.56250916
C	8.89116877	-0.60419872	0.00132622
C	7.77111374	-1.29068959	0.52507486
C	6.51516848	-0.72406294	0.49128238
C	6.32702914	0.57822475	-0.07133053
O	5.466346	-1.39570126	1.06494992
C	-3.03357543	-0.59793992	-1.59543333
C	-4.99099379	5.14011684	0.52627807
O	-2.58339507	4.35467632	-0.98947343

C	-2.441419	-3.35037271	-0.36428933
O	-1.36636254	-2.90602869	0.31555401
O	-2.34123761	-3.96099797	-1.41259696
C	-0.07990977	-3.05954346	-0.3126638
C	0.94124118	-2.68559193	0.74767971
O	2.17364296	-2.46602895	0.08672153
C	3.22532839	-2.15588599	0.99026039
C	4.46334114	-1.87781481	0.15725543
C	-3.68672585	-3.7826672	1.72310238
C	-3.84862294	1.49291985	0.83840764
C	-4.75143259	0.76321732	-0.20108568
C	-4.63401659	2.71570539	1.37641984
C	-3.47545277	0.55281532	1.98630791
C	-2.63918215	2.20872825	0.18229974
C	-4.26331529	-0.65710961	-0.66612733
C	-5.09829211	1.71181373	-1.36728167
C	-4.6247197	3.69894926	0.19876377
C	-2.94305394	-0.79928221	1.519575
C	-3.18549803	3.54689371	-0.30602693
C	-3.97469747	-1.49790181	0.62011435
C	-5.43656015	-1.3187778	-1.4335126
C	-5.54424184	3.10790486	-0.90197197
C	-3.74384333	-3.03070611	0.37174904
C	-5.23765664	-2.80997132	-1.71236201
C	-4.93734552	-3.58726028	-0.43176022
H	-5.69033578	0.56597902	0.33788472
H	-4.91918998	-1.48938972	1.18655407
H	3.93009596	2.75082779	-0.58432868
H	5.7979236	4.06842765	-1.57801025
H	8.10015727	3.08535352	-1.58214628
H	9.59949612	1.17327739	-0.97180649
H	9.86768844	-1.07545734	0.04268704
H	7.88180318	-2.27603371	0.96474326
H	-2.85754456	-1.55994641	-2.07724784
H	-3.18124617	0.12687241	-2.39938877
H	-2.11056286	-0.33369313	-1.08042443
H	-6.0435482	5.21552258	0.81686312
H	-4.38152276	5.52223712	1.35154313
H	-4.82612033	5.78294825	-0.34332365
H	0.0457973	-4.08425633	-0.66713853
H	-0.01218255	-2.3745564	-1.16117751
H	0.61143728	-1.77314097	1.26438011
H	1.03509866	-3.48131056	1.50061435
H	3.39930823	-2.99018144	1.68528996

H	2.98185359	-1.26453061	1.58406026
H	4.23506741	-1.12826765	-0.60230606
H	4.82000017	-2.7896975	-0.33788012
H	-2.80037471	-3.52670541	2.30356828
H	-4.57498011	-3.54116313	2.3147339
H	-3.67785702	-4.86380865	1.55182465
H	-5.6467249	2.45063006	1.69700954
H	-4.10888034	3.14222741	2.24107282
H	-2.74284306	1.04560516	2.63511058
H	-4.37521579	0.38429505	2.59470718
H	-2.13966287	1.68282626	-0.63165225
H	-1.88130227	2.42529459	0.94636467
H	-5.88602245	1.27891918	-1.98869916
H	-4.22832251	1.82646361	-2.02272697
H	-1.99573245	-0.67040109	0.99238305
H	-2.71757823	-1.41572844	2.39312853
H	-6.35241155	-1.19047649	-0.83997652
H	-5.60194921	-0.79491403	-2.38041899
H	-5.57386275	3.78873447	-1.75995403
H	-6.55911808	3.06332001	-0.4874962
H	-6.14325213	-3.21359577	-2.17995997
H	-4.42621363	-2.96392527	-2.4284219
H	-5.8143826	-3.54680014	0.22685277
H	-4.75715288	-4.64241969	-0.65848103
C	1.80868231	0.57328653	-0.20852747
C	0.81100608	0.81545277	0.64720629
C	1.08523104	1.22354551	2.04351311
O	2.18101297	1.25218714	2.57150844
O	-0.05410583	1.56020108	2.68409667
C	1.50503136	0.13508479	-1.59124366
O	0.3924554	-0.10522513	-2.02735261
O	2.62316245	0.03238595	-2.33236888
H	2.85244175	0.67547887	0.07803374
H	-0.22279737	0.71934024	0.34029479
H	0.18018247	1.8170928	3.59286602
H	2.36072933	-0.27271961	-3.21844246

Table S5. HOMO and LUMO Energy Gap of 5, 5-MA and 5-FA.

	5	5-MA	5-FA
HOMO	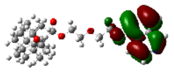	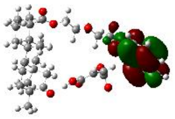	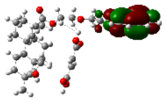
LUMO	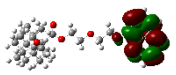	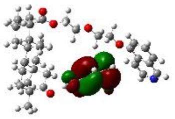	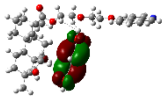
E_h	-5.859426455eV	-5.912488646eV	-5.889903201eV
E_l	-1.318663475eV	-1.546422725eV	-2.508889236eV
ΔE	4.540762981eV	4.366065921eV	3.381013965eV

Table S6. Cartesian coordinates of the optimized geometry of 5 in the solvent phase.

		**5**	
C	7.67341095	1.6971336	1.20946614
C	8.65291249	2.52016953	1.71759385
N	9.93802272	2.543514	1.26320318
C	10.24536939	1.72208747	0.27694709
C	9.33425947	0.8210753	-0.33945711
C	9.71439754	-0.04433199	-1.3994352
C	8.77972701	-0.88988456	-1.94797939
C	7.44238217	-0.91585234	-1.47657044
C	7.04999983	-0.08258924	-0.44605686
C	7.99510765	0.81263696	0.15098496
O	5.79533063	-0.02888151	0.07946314
C	-3.35565643	-0.67289552	-1.50422452
C	-6.64490364	4.68389894	0.03741707
O	-4.21308141	4.35098368	-1.60391795
C	-1.94061431	-2.9901571	0.07675565
O	-0.95164804	-2.16122789	0.47863531
O	-1.77041979	-3.81070525	-0.80638436
C	0.3117676	-2.29927416	-0.20132912
C	1.26776124	-1.32147197	0.46168204
O	2.51774021	-1.46826976	-0.19078185
C	3.51823188	-0.6109957	0.33005699
C	4.79102947	-0.89123792	-0.45433458

C	-2.86163699	-3.28374069	2.34424317
C	-4.49961158	1.56246109	0.7053426
C	-5.24797502	0.45541676	-0.1107718
C	-5.55569811	2.60780141	1.16063513
C	-3.79002732	0.95153165	1.9207122
C	-3.58964446	2.46071301	-0.17945739
C	-4.43884257	-0.86709704	-0.41772291
C	-5.92927338	1.0782791	-1.35221261
C	-5.90980622	3.3577717	-0.13417063
C	-2.94788957	-0.28359354	1.59202555
C	-4.52148981	3.51991544	-0.7690386
C	-3.8240782	-1.36860081	0.93781284
C	-5.45913204	-1.92920455	-0.91713484
C	-6.70463934	2.37231768	-1.0368055
C	-3.22252775	-2.82449872	0.90605636
C	-4.90301801	-3.35628668	-0.97824338
C	-4.30324181	-3.78865408	0.36128623
H	-6.05726606	0.12424356	0.55822306
H	-4.68800703	-1.49247099	1.6106583
H	6.66747813	1.72216205	1.61101896
H	8.42670768	3.20632639	2.53005616
H	11.27480562	1.74612755	-0.08130627
H	10.73737003	-0.0270522	-1.7626479
H	9.05564077	-1.5569417	-2.75881057
H	6.73770641	-1.59734545	-1.93684727
H	-2.93411908	-1.629346	-1.81995585
H	-3.77564362	-0.21122868	-2.4018553
H	-2.52253665	-0.05183016	-1.16972699
H	-7.6537209	4.52159812	0.4308431
H	-6.11287809	5.34355989	0.73111194
H	-6.73023808	5.20215229	-0.92254141
H	0.67439168	-3.32625537	-0.10778262
H	0.18931339	-2.07183822	-1.26390254
H	0.89057951	-0.29248788	0.3630873
H	1.35448906	-1.54662324	1.53528253
H	3.68743813	-0.80364597	1.40004718
H	3.2325976	0.445694	0.21843396
H	4.63338508	-0.68687659	-1.52029658
H	5.08327594	-1.94197804	-0.33890567
H	-2.02319006	-2.72622851	2.76367108
H	-3.72814932	-3.15694734	3.0009799
H	-2.59288481	-4.34533574	2.34418619
H	-6.42819439	2.14139305	1.63120279
H	-5.10886962	3.29253096	1.89435385

H	-3.17156795	1.71848264	2.4045715
H	-4.55588919	0.66536858	2.65615321
H	-3.02374261	1.96502077	-0.96969385
H	-2.85922721	2.98666348	0.4513555
H	-6.61714274	0.36168377	-1.80988092
H	-5.17777821	1.30140152	-2.11827246
H	-2.11060059	-0.01194786	0.9434648
H	-2.50251554	-0.66120545	2.51672214
H	-6.32615499	-1.92117203	-0.24138945
H	-5.83505575	-1.64778694	-1.9068639
H	-6.97989866	2.87102903	-1.97353483
H	-7.63947609	2.134691	-0.51274835
H	-5.71051315	-4.04510606	-1.2546339
H	-4.14552023	-3.4443355	-1.76303806
H	-5.10189835	-3.83544873	1.113329
H	-3.88193238	-4.79554438	0.28290752

Table S7. Cartesian coordinates of the optimized geometry of 5-MA in the solvent phase.

	**5-MA**		
C	6.36084267	0.14640607	1.41579767
C	7.26329769	1.059546	1.91103616
N	8.41334146	1.41544028	1.271866
C	8.66104377	0.84612781	0.10728777
C	7.81275063	-0.10354386	-0.52314388
C	8.1077997	-0.67436	-1.78849255
C	7.22760727	-1.56972698	-2.34799007
C	6.03053621	-1.93857148	-1.68408192
C	5.7309977	-1.40338933	-0.44697035
C	6.61858803	-0.46410122	0.1649572
O	4.61115566	-1.67915699	0.27261933
C	-3.37719553	-0.75359825	-1.40006514
C	-3.99455343	5.41490336	0.19083173
O	-1.85589098	3.92909243	-1.1925614
C	-3.40264808	-3.44834622	-0.00223639
O	-2.24502611	-3.08661617	0.58529808
O	-3.43871557	-4.21653947	-0.9451599
C	-1.02390319	-3.51216742	-0.04364816
C	0.07579527	-2.74884568	0.67164758
O	1.29334199	-3.01375342	0.00196154
C	2.38256052	-2.33878857	0.61006116
C	3.5552787	-2.4093249	-0.3510927
C	-4.70178424	-3.48223958	2.0886397
C	-3.77425072	1.64454663	0.85263352
C	-4.78825997	1.05032404	-0.17088797

C	-4.26142393	3.05900097	1.25314878
C	-3.66346891	0.74625332	2.08422284
C	-2.41413792	1.99539965	0.19657938
C	-4.6089618	-0.47488846	-0.51197598
C	-4.8863614	1.95265482	-1.41940523
C	-3.98360372	3.90351621	0.00088187
C	-3.40964122	-0.71819091	1.73972477
C	-2.6307383	3.3560087	-0.43136745
C	-4.53212717	-1.25834496	0.83899595
C	-5.88250432	-0.93045975	-1.27026242
C	-4.99650616	3.44983417	-1.08740424
C	-4.61096417	-2.81880132	0.6934765
C	-6.00445301	-2.44763835	-1.43247662
C	-5.89044114	-3.17283743	-0.09212399
H	-5.76079335	1.10572103	0.33997652
H	-5.47158729	-1.02066282	1.36223356
H	5.45905812	-0.09673378	1.96320119
H	7.08268536	1.54725284	2.86530912
H	9.5819404	1.13638748	-0.39801338
H	9.02149396	-0.39115564	-2.30094737
H	7.43890161	-2.00996627	-3.31699699
H	5.36060998	-2.64607438	-2.15637234
H	-3.41073776	-1.76175405	-1.81432025
H	-3.34032292	-0.07328283	-2.25339757
H	-2.43524901	-0.66288085	-0.85816782
H	-5.00118273	5.75879036	0.44669727
H	-3.31661339	5.71371763	0.99632483
H	-3.68004622	5.92079559	-0.72630181
H	-0.90369999	-4.59402886	0.05644623
H	-1.0474275	-3.25967245	-1.10697808
H	-0.15137352	-1.67212492	0.64930014
H	0.12640525	-3.05547297	1.72703137
H	2.64890245	-2.80496599	1.57042763
H	2.14084772	-1.28667586	0.80628502
H	3.2851311	-1.94616435	-1.30752041
H	3.85652724	-3.44723486	-0.53556455
H	-3.77589815	-3.38230088	2.65602384
H	-5.5139293	-3.02425792	2.66137335
H	-4.92062635	-4.54937345	1.98310815
H	-5.31751448	3.07200882	1.53908825
H	-3.67655338	3.42551365	2.1062453
H	-2.87342289	1.12884637	2.74189244
H	-4.60365942	0.8226502	2.64782557
H	-2.02032515	1.28970563	-0.53392047

H	-1.63563394	2.12070092	0.9610158
H	-5.74486537	1.6635205	-2.03010167
H	-4.00572393	1.80562091	-2.05379084
H	-2.43691526	-0.8229406	1.25618182
H	-3.35209982	-1.298822	2.66366202
H	-6.759346	-0.56378382	-0.7192063
H	-5.91484182	-0.4596037	-2.25804895
H	-4.84575321	4.0404598	-1.99729465
H	-5.9973269	3.68496004	-0.70635754
H	-6.96971301	-2.68368342	-1.89488078
H	-5.23784536	-2.82563725	-2.11466828
H	-6.74402193	-2.8998726	0.54121109
H	-5.93432618	-4.25577596	-0.24015141
C	2.26736657	1.4778475	-1.30115729
C	3.2685732	1.1988663	-0.46342086
C	3.3009977	1.55550998	0.99015362
O	2.93048317	0.81613216	1.87988774
O	3.8979723	2.73776747	1.2036458
C	1.07558339	2.25611848	-0.87743186
O	0.8875591	2.63993014	0.27023142
O	0.24806803	2.49038766	-1.893601
H	2.30947986	1.15745546	-2.33621242
H	4.13851537	0.65267187	-0.81325236
H	3.93560274	2.87649042	2.16707961
H	-0.52184073	3.05740465	-1.58375536

Table S8. Cartesian coordinates of the optimized geometry of 5-FA in the solvent phase.

	**5-FA**		
C	-8.1686626	0.40707775	1.30726307
C	-9.35252386	0.24176629	1.98902327
N	-10.41951189	-0.45297181	1.50205089
C	-10.29455568	-0.99352422	0.30479484
C	-9.1310445	-0.90082653	-0.50527084
C	-9.04348387	-1.5007822	-1.78832769
C	-7.88565516	-1.36987891	-2.51664182
C	-6.77567657	-0.64685102	-2.01162224
C	-6.84056418	-0.05602989	-0.76529612
C	-8.02777894	-0.16972577	0.02273141
O	-5.83537257	0.65744558	-0.18785718
C	3.50444678	1.44196408	-1.6294538
C	6.48934918	-4.10986833	-0.42002117
O	4.38412239	-3.41108811	-2.36749877
C	1.8733134	3.51029982	-0.05638145
O	0.8811691	2.60186857	0.06415899

O	1.83178288	4.43653518	-0.84355286
C	-0.25181361	2.68173089	-0.81887541
C	-1.50372222	2.53916605	0.04152698
O	-2.46071821	1.77340733	-0.70625571
C	-3.69782314	1.61649046	-0.00615742
C	-4.61486336	0.80601128	-0.90540191
C	2.36934419	3.52049584	2.35355781
C	4.31036998	-1.06250419	0.38818086
C	5.17379813	0.12560993	-0.13137831
C	5.26805854	-2.19626562	0.8324909
C	3.43901008	-0.6125322	1.5613211
C	3.54253025	-1.78114381	-0.75096371
C	4.4101702	1.47821566	-0.38037873
C	6.04340675	-0.3236022	-1.32402333
C	5.81417235	-2.74720033	-0.49090961
C	2.63520483	0.65107823	1.26925668
C	4.55053877	-2.74950434	-1.35905717
C	3.58996238	1.802739	0.9105436
C	5.48307694	2.58065398	-0.57509983
C	6.75451985	-1.66623622	-1.0854679
C	2.98642965	3.24963336	0.95995447
C	4.93025219	4.00684176	-0.5376663
C	4.12551199	4.26513492	0.73492475
H	5.86253355	0.35028213	0.69686259
H	4.33349192	1.83187012	1.7222773
H	-7.3518465	0.96961926	1.74234113
H	-9.48270586	0.67578746	2.9768579
H	-11.15118352	-1.54756008	-0.07857155
H	-9.8919027	-2.05486615	-2.17682118
H	-7.80405984	-1.82298625	-3.4992628
H	-5.88231578	-0.56719572	-2.61778527
H	3.17050308	2.44383159	-1.90248474
H	4.04087236	1.04848402	-2.49566941
H	2.60969229	0.83512937	-1.48559938
H	7.42097975	-4.05174342	0.15159459
H	5.83767068	-4.84481266	0.0635092
H	6.72300142	-4.47297879	-1.42505723
H	-0.21905686	3.61673671	-1.37608691
H	-0.17799054	1.84330447	-1.51656666
H	-1.25309166	2.00666913	0.96483952
H	-1.93621339	3.5134386	0.2947501
H	-4.13819255	2.59889689	0.2005562
H	-3.52947366	1.09276661	0.94184925
H	-4.17447743	-0.17557702	-1.12025256

H	-4.7820884	1.32896158	-1.85426285
H	1.4797249	2.91753874	2.53737166
H	3.10804676	3.30013164	3.13007108
H	2.088649	4.57470667	2.44197475
H	6.05872934	-1.83665424	1.49894501
H	4.70184454	-2.96929702	1.36861062
H	2.77621393	-1.43062929	1.86279895
H	4.09721913	-0.42096121	2.42041197
H	3.09688482	-1.1507017	-1.51947211
H	2.7401622	-2.39815087	-0.33186652
H	6.79304131	0.43695023	-1.55580444
H	5.42251764	-0.41632653	-2.2214973
H	1.92285335	0.46638665	0.4622224
H	2.03674806	0.90847657	2.14648311
H	6.23334694	2.4740422	0.22039247
H	6.00812338	2.4227181	-1.52260592
H	7.18011462	-2.02798767	-2.02805561
H	7.58644237	-1.53570413	-0.38217452
H	5.76237601	4.71792048	-0.59526052
H	4.30025855	4.2004338	-1.41029409
H	4.79247674	4.19996361	1.60393762
H	3.70938531	5.27668848	0.72561921
C	-0.06198423	-2.18170902	0.3674206
C	0.39409982	-2.67053227	1.52475648
C	1.32101119	-3.8267902	1.54954296
O	1.72617322	-4.42558291	0.56897897
O	1.69895448	-4.12403764	2.80652794
C	-0.93753324	-0.98148629	0.34446019
O	-1.34616289	-0.41442656	1.34715686
O	-1.20719071	-0.58920446	-0.9032228
H	0.21601559	-2.62984019	-0.5801998
H	0.11676152	-2.21482269	2.46877228
H	2.31837011	-4.87337475	2.75761859
H	-1.74128479	0.25440768	-0.86698745

References

1. Frisch, M.J.; Trucks, G.W.; Schlegel, H.B.; Scuseria, G.E.; Robb, M.A.; Cheeseman, J.R.; Scalmani, G.; Barone, V.; Petersson, G.A.; Nakatsuji, H.; et al. *Gaussian 16 Rev. B.01*, Wallingford, CT, 2016.
